# High‐Entropy Materials for Water Splitting: An Atomic Nanoengineering Approach to Sustainable Hydrogen Production

**DOI:** 10.1002/adma.202506117

**Published:** 2025-06-16

**Authors:** Yufei Zhao, Jinhu Wu, Xianjun Cao, Dongfang Li, Peng Huang, Hong Gao, Qinfen Gu, Jinqiang Zhang, Guoxiu Wang, Hao Liu

**Affiliations:** ^1^ Joint International Laboratory on Environmental and Energy Frontier Materials School of Environmental and Chemical Engineering Shanghai University Shanghai 200444 China; ^2^ School of Mathematical and Physical Sciences Faculty of Science University of Technology Sydney Broadway Sydney NSW 2007 Australia; ^3^ School of Chemistry and Materials Science Jiangsu Normal University Xuzhou Jiangsu 221116 China; ^4^ Australian Synchrotron ANSTO 800 Blackburn Rd Clayton VIC 3168 Australia

**Keywords:** element roles, high entropy materials, structural evolution, water splitting

## Abstract

Green hydrogen production via water electrolysis is pivotal for achieving energy sustainability. However, the inherently sluggish kinetics of the hydrogen evolution reaction and oxygen evolution reaction impede the progress of water‐splitting technology. Recently, high‐entropy materials (HEMs) composed of at least five elements have garnered significant attention as promising electrocatalysts for water splitting, owing to their compositional versatility, structural robustness, and synergistic interactions among elements. This review comprehensively explores the development of HEMs, tracing their emergence and structural evolution via atomic nanoengineering strategies (i.e., from bulk to nanostructuring, from random distributions to relatively ordered architectures, from bare HEMs to reconstructed HEMs, from intact HEMs to defective structures, from pristine HEMs to functionalized variants) and revealing how these evolutionary steps contribute to the properties and enhance catalytic performance in water splitting. The fundamental roles of individual elements (e.g., active sites, promoters, stabilizers) in shaping the structure, stability, and catalytic activity of HEMs are examined, laying a foundation for the rational design of efficient HEM‐based electrocatalysts. The review also highlights recent advances in HEM‐based catalysts for water splitting, emphasizing desirable properties and elemental contributions. Finally, the remaining challenges and perspectives on the future directions of HEM‐based materials in energy conversion technologies are discussed.

## Graphical abstract

High‐entropy materials (HEMs), typically composed of five or more principal elements in near‐equimolar ratios, offer significant potential due to their unique properties. This review outlines the emergence and structural evolution of HEMs, followed by an in‐depth discussion of the elemental roles (active sites, promoters, or stabilizers) for water splitting. Last, future research directions in this evolving field are discussed.

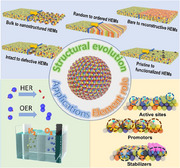

## 1 Introduction

Hydrogen is a clean and sustainable energy resource with significant potential to replace fossil fuels. It is considered a promising renewable energy carrier due to its exceptionally high mass‐energy density, making it an attractive alternative to conventional fuels.^[^ ^1^, ^2^, ^3^, ^4^ ^]^ However, the large‐scale production of hydrogen is still limited by its synthesis approach that relies heavily on steam reforming of natural gas, inevitably accompanied by emissions of carbon dioxide.^[^ ^5^, ^6^, ^7^ ^]^ Water splitting, on the other hand, is an alternative way to generate hydrogen with zero carbon emissions.^[^ ^8^, ^9^, ^10^, ^11^ ^]^ Nevertheless, the widespread application of water electrolysis is hindered by its reliance on highly efficient catalysts, which are currently based on precious metals such as platinum (Pt), ruthenium (Ru), and iridium (Ir).^[^ ^12^ ^]^ The high cost and scarcity of these precious metal‐based catalysts significantly restrict the scalability of water‐splitting electrolyzers. Therefore, developing active and cost‐effective catalysts for water‐splitting systems is critical for potential large‐scale production of hydrogen.

Transition metal‐based electrocatalysts, such as sulfides, phosphides, carbides, nitrides, oxides, alloys, etc., have drawn intensive attention in the past few decades.^[^ ^13^, ^14^, ^15^, ^16^, ^17^, ^18^ ^]^ However, they face limitations in stability, conductivity, selectivity, and scalability, which hinder their long‐term efficiency and industrial applicability in water splitting. High‐entropy materials (HEMs), defined by comprising typically five principal elements or more in near‐equimolar ratios, possess significant potential owing to the highly disordered homogeneous single‐phase and high configurational entropy of mixing.^[^ ^19^, ^20^, ^21^ ^]^ The development of HEMs can be traced back to the early 2000s with two seminal papers published on metallic alloys containing five or six principal components.^[^ ^22^, ^23^ ^]^ These alloys quickly attract attention within the materials science community due to their core effects, including the high‐entropy effect, lattice distortion effect, and cocktail effect. Specifically, the high‐entropy effect refers to the thermodynamic stabilization resulting from the large configurational entropy generated by mixing multiple principal elements. The lattice distortion effect arises from atomic size mismatches and complex chemical interactions among the constituent elements, leading to local lattice strain. Meanwhile, the cocktail effect describes the unexpected synergies and emergent properties that result from the intricate interactions among the diverse elements within HEMs.^[^ ^24^, ^25^, ^26^, ^27^ ^]^ The family of HEMs has expanded from HEAs to high entropy oxides (HEOs), high entropy carbides (HECs), high entropy nitrides (HENs), high entropy sulfides (HESs), high entropy borides (HEBs), etc.^[^ ^28^, ^29^, ^30^, ^31^, ^32^, ^33^, ^34^, ^35^, ^36^ ^]^ The random distribution of multiple metal elements in HEMs promotes exceptional homogeneity, potentially surpassing the properties of single or unary element‐based materials. HEMs leverage synergistic interactions (the combined effect of multiple components is greater than the sum of their individual effects) among multiple principal elements, leading to a higher density of active sites, optimized electronic structures, and superior structural integrity.^[^ ^37^, ^38^, ^39^ ^]^ These unique features enhance electrochemical activity and ensure outstanding stability for water‐splitting applications.^[^ ^40^, ^41^ ^]^

With continuous advancements in nanotechnology, HEMs have undergone significant structural evolution, leading to substantial enhancements in their electrocatalytic activity. Unlike conventional alloys or metal compounds, HEMs possess a diverse range of unique surface binding sites, providing a versatile platform for precisely tuning binding energies to optimize reaction properties.^[^ ^42^ ^]^ This ability is crucial for water splitting which relies significantly on the interaction of active sites with reactants and intermediates.^[^ ^43^, ^44^ ^]^ In particular, the structural evolution of HEMs plays a vital role in tailoring the geometric and electronic features to modulate the adsorption energy, thereby enhancing their catalytic efficiency for water splitting.^[^ ^45^, ^46^ ^]^ The selection and composition of elements in HEMs influence both the density and local environment of active sites, thereby directly impacting the intrinsic catalytic activity.^[^ ^47^, ^48^, ^49^ ^]^ Furthermore, downsizing HEMs from bulk to the nanoscale and engineering porous or hierarchical nanostructures can dramatically increase surface area, facilitate electron and mass transport, and improve atomic utilization, all of which contribute to accelerated reaction kinetics and enhanced catalytic performance.^[^ ^50^, ^51^, ^52^, ^53^, ^54^ ^]^ Another critical structural advancement involves the transition from a randomly distributed atomic arrangement to a relatively ordered configuration, which further optimizes HEMs for water‐splitting applications.^[^ ^55^ ^]^ Other than the structure evolution for the pristine HEMs, surface engineering approaches, including creating reconstructed, defective, or functionalized HEMs, offer additional pathways for expanding and refining their properties.^[^ ^56^, ^57^, ^58^ ^]^ These surface modifications can introduce new active sites, enhance stability, and further improve catalytic performance, making HEMs increasingly attractive as next‐generation electrocatalysis.

**Figure 1:**
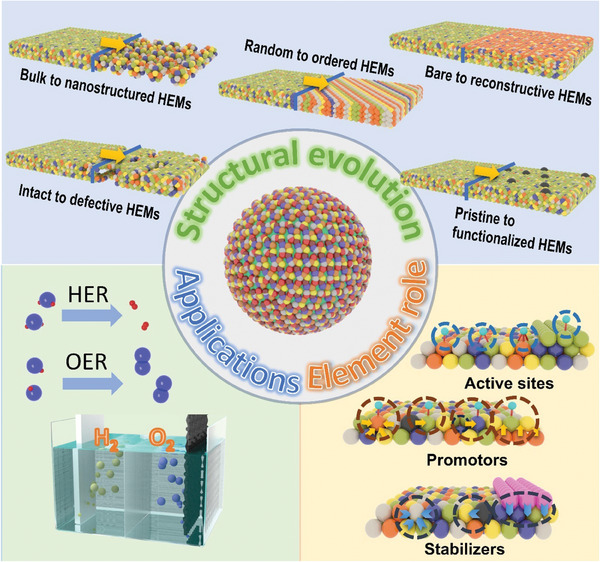
Overview of structural evolution and elemental roles of HEMs for water splitting.

Several reviews have summarized the synthesis methods, properties, effects, design strategies, and potential applications of HEMs.^[^ ^59^, ^60^, ^61^, ^62^, ^63^ ^]^ However, a comprehensive review detailing the evolution of HEMs from structural foundations to functionality, as well as an in‐depth understanding of the roles each element plays, is still lacking. This updated review focuses on recent advancements in HEMs for water splitting. In this review, we will discuss the emergency and structural evolution of HEMs, covering the structural progression from bulk forms to nanostructured, from random atomic distributions to more ordered structures, from bare HEMs to reconstructed, from intact to defective structures, and from pristine HEMs to functionalized HEMs, all of which significantly enhance their properties for water splitting applications. We then elaborate on how each element contributes to overall structure, stability, and performance to identify elemental roles in HEMs, providing a foundation for designing efficient HEM‐based electrocatalysts. This review will also summarize HEM‐based catalysts with desired properties and identified elemental roles for both hydrogen evolution reaction (HER) and oxygen evolution reaction (OER) in water splitting. Finally, we address current challenges and future perspectives for HEM‐based catalysts in energy conversion applications (**Figure** **1**).

## 2 The Emergence and Structure Evolution of High Entropy Materials

### 2.1 The Emergence of High Entropy Materials

**Figure 2:**
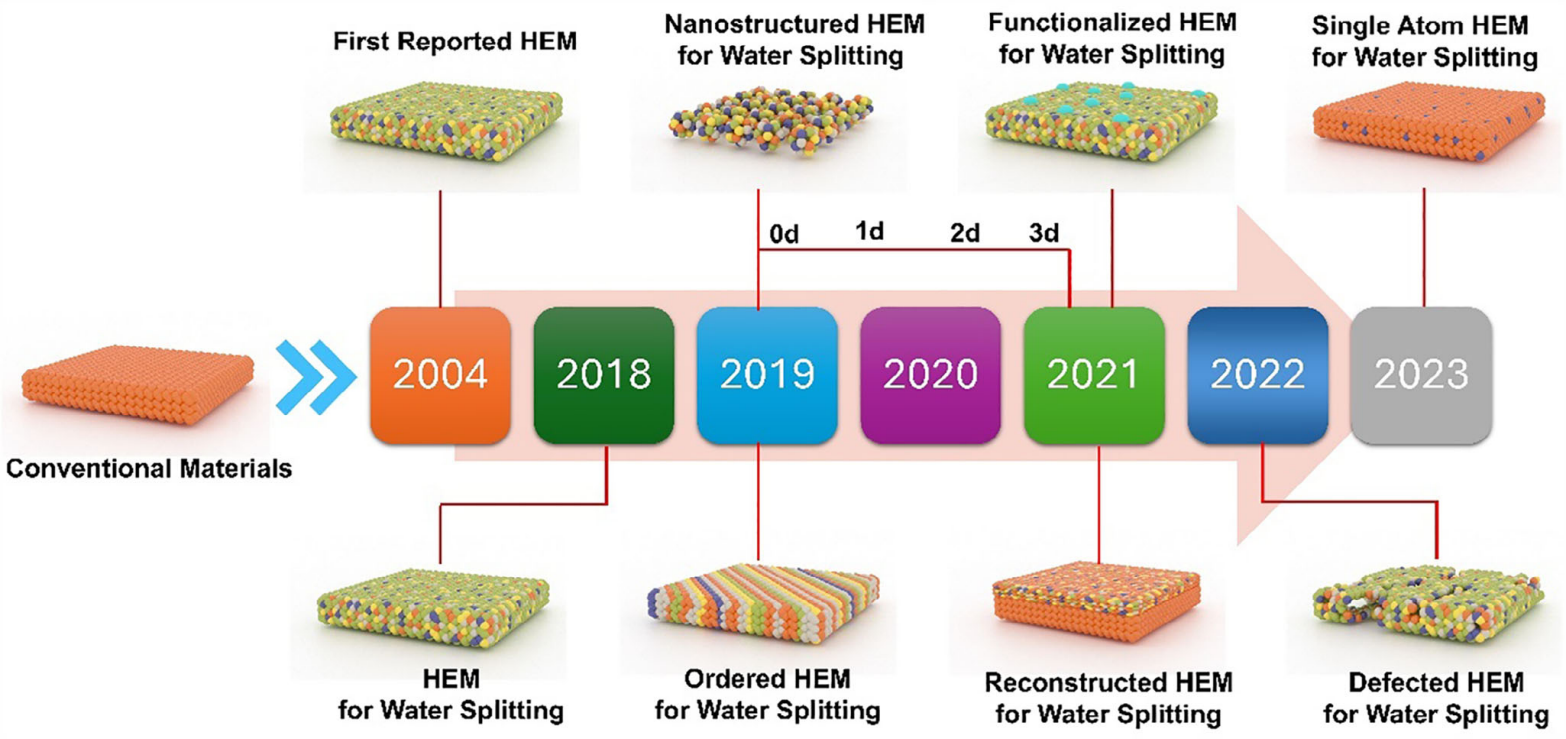
Historical timeline of emergency and structural evolution of HEMs for water splitting.

The development from non‐high‐entropy materials to HEMs marked the emergence of a new design paradigm, which leverages multiple principal elements (≥5) in near‐equimolar ratios to harness configurational entropy and unlock unique material properties (**Figure** **2**).^[^ ^64^, ^65^, ^66^, ^67^, ^68^, ^69^ ^]^ This transition reflects a shift from simplicity and predictability to controlled complexity and tunability, opening a new frontier in functional materials design. HEMs resolve the limitation of the stability, corrosion resistance, and versatility in traditional non‐high‐entropy materials, displaying enhanced stability, chemical properties, cocktail effects, etc., making them promising for water splitting.^[^ ^70^, ^71^ ^]^

Generally, the simplest nanocrystals are monometal‐based compounds, consisting of a uniform arrangement of atoms from a single metallic element.^[^ ^72^, ^73^, ^74^, ^75^ ^]^ Their properties are primarily governed by the intrinsic nature of the metal, crystal structure, and particle size. Their relatively simple composition allows for precise control over their synthesis and the investigation of structure–catalytic properties. Advancements in alloying and doping strategies with additional metal elements further enable precise engineering of the properties of monometal‐based structures, which typically consist of two or three compositional elements with one primary component.^[^ ^76^, ^77^, ^78^ ^]^ This strategy has facilitated the development of numerous materials based on a primary component coupled with additional minor elements improved conductivity and catalytic activity. For instance, we have designed Ni‐doped CoP and Co‐doped MnO_2_ for water splitting, effectively modulating the electronic structures of the primary metals, namely Co and Mn, respectively, to significantly enhance their catalytic performance.^[^ ^79^, ^80^ ^]^ Although proven effective, these strategies often lead to localized effects, and the central properties of the multicomponent phase space may deviate significantly from the corresponding corners and edges. Moreover, the doped metal compounds often suffer from dopant leaching or phase segregation under harsh reaction conditions.

Further development of metal alloys or compounds has progressed from simple monometal‐based compounds or doped materials to more complex systems (e.g., ternary or quaternary), allowing for increased tunability of composition, structure, and functionality.^[^ ^81^ ^]^ For instance, Ni–Fe oxide aerogels synthesized via a modified epoxide route exhibited high catalytic activity for OER, achieving 10 mA cm^−2^ at an overpotential of 380 mV with an optimized Ni/Fe ratio.^[^ ^82^ ^]^ Similarly, modulated NiFeX and FeCoX (*X* = W, Mo, Nb, Ta, and Re) oxyhydroxide catalysts demonstrated excellent OER performance, attributed to the facilitated oxidation of 3d transition metals induced by the incorporation of high‐valence modulators.^[^ ^83^ ^]^ This systematic progression toward multi‐metallic systems paves the way for the design of HEMs, which composed of multiple principal elements in nearly equiatomic proportions. HEMs exhibit significantly higher configurational entropy, enhanced multi‐element synergy, superior structural stability, and tunable electronic properties, offering unconventional compositions with many possibilities to regulate catalytic performance and overcome the limitations of single‐element or doped catalysts. These enable them to be a highly promising alternative for water splitting. Various HEMs have been designed and prepared, for example, PtCoNiRuIr HEA‐NPs, PtRuMoFeCoNi ultra‐small HEA quantum dots, PtRuPdCoNi HEA nanoparticles, M‐RuIrFeCoNiO_2_, have exhibited unique properties and excellent HER/OER performance.^[^ ^84^, ^85^, ^86^, ^87^ ^]^ Further to precious metal‐based HEMs, non‐precious metal‐derived HEMs, composed of multiple inexpensive elements, offer cost‐effective alternatives while maintaining exceptional catalytic performance. HEMs such as CoFeMnCuZn, FeCoMoW, and Co_0.6_(VMnNiZn)_0.4_PS_3_ nanosheets, with high‐concentration active sites, have been successfully developed, exhibiting enhanced HER/OER performance.^[^ ^88^, ^89^, ^90^ ^]^

### 2.2 The Structure Evolution of High Entropy Materials

Since the first appearance of HEMs, the structural evolution of HEMs plays a crucial role in expanding their structural, electronic, and catalytic properties, significantly broadening their potential applications. Typically, several significant steps have marked the evolution of HEMs. The first is to introduce nanostructures to the bulky HEMs which can significantly enhance the surface‐to‐volume ratio, exposing a greater density of active sites and facilitating improved mass transport.^[^ ^91^, ^92^, ^93^ ^]^ This evolution optimizes charge transfer dynamics and catalytic efficiency, leading to superior performance for water splitting. Another structural evolution is from the random distribution of metal elements in the crystal lattice to a relatively ordered structure in HEMs which can maintain entropy stabilization from inherent disorder while generating unique electronic properties from controlled structural ordering. Moreover, the development of reconstructed, defective, and functionalized HEMs represents a significant advancement, compared to the pristine structures, to further optimize the properties and catalytic capabilities of HEMs. Thus, in this section, we will focus on the critical steps of the structural evolution in HEMs with an in‐depth understanding of properties and optimized catalytic performance, namely from bulk to nanostructuring, from random distribution to relatively ordered structure, from pristine HEMs to their reconstructed, defect‐engineered, and functionalized forms.

#### 2.2.1 From Bulk to Nanostructuring

**Figure 3:**
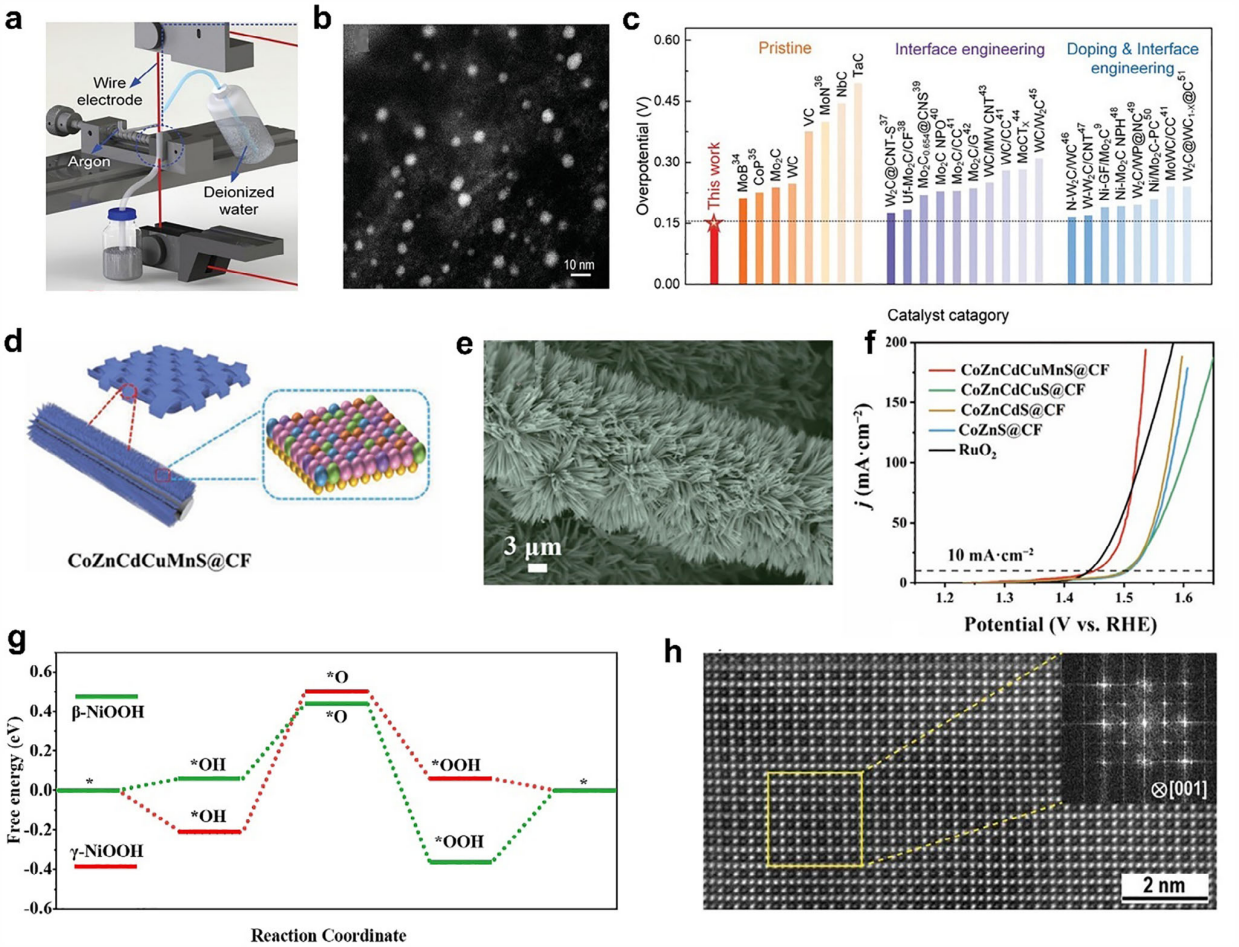
a) Schematic process of proceeding HEC bulk to nanoparticles by WEDM. b) High‐Angle Annular Dark‐Field Scanning Transmission Electron Microscopy (HAADF‐STEM) image of (MoWVNbTa)C HECNPs. c) Comparison of η_10_ between the HECNPs and other nonprecious metal‐based catalysts. a–c) Reproduced with permission.^[^ ^100^ ^]^ Copyright 2022, Wiley‐VCH. d–f) schematic diagram, Scanning Electron Microscope (SEM) and OER polarization curves of the synthesis process of CoZnCdCuMnS@CF. d–f) Reproduced with permission.^[^ ^114^ ^]^ Copyright 2022, Springer Nature. g) OER reaction energy diagram on β‐NiOOH and γ‐NiOOH models. g) Reproduced with permission.^[^ ^120^ ^]^ Copyright 2022, Elsevier. h) Aberration‐corrected HAADF‐STEM image viewed along the [001] zone axis. h) Reproduced with permission.^[^ ^43^ ^]^ Copyright 2020, Wiley‐VCH.

While most studies of HEMs mainly focus on adjusting the chemical composition to optimize the catalytic performance, structural engineering, especially the regulation of size and dimensionality, has also emerged as a critical approach to improve catalytic efficiency. HEMs are predominantly synthesized as bulk materials using conventional methods (e.g., melt processing), due to the challenges associated with mixing elements with vastly different chemical and physical properties, as well as cooling rate constraints.^[^ ^22^, ^94^ ^]^ Reducing the size of HEMs to the nanoscale with controlled morphologies is significant for enhancing nanostructural diversity and investigating relationships between structures and properties. The typical low‐dimensional HEMs are nanoparticles, which feature small size and high specific surface area, exposing abundant active sites and maximizing atom utilization.^[^ ^95^, ^96^, ^97^, ^98^, ^99^ ^]^ A typical example is the well‐dispersed sub‐10 nm (MoWVNbTa)C HEC nanoparticles, which were successfully synthesized via electrical discharge‐induced bulk‐to‐nanoparticle transformation (**Figure** **3**a,b). These HEC nanoparticles have demonstrated outstanding catalytic activity and long‐term durability for HER, which was attributed to their unique microstructure and the enhanced electronic interactions arising from high configurational entropy (Figure 3c).^[^ ^100^ ^]^ Similarly, PdCuPtNiCo HEA nanoparticles were synthesized using PdCu nanoparticles as seeds to initially form PdCu/PtNiCo core–shell structures, followed by an annealing process.^[^ ^101^ ^]^ Generally, substrates are critical to controlling the size of HEM nanoparticles. The commonly used substrates are carbon nanotubes, graphene, and organic materials‐derived carbon substrates, among others.^[^ ^102^, ^103^ ^]^ A carbon shock method has been developed to synthesize HEA and HEO nanoparticles uniformly dispersed on carbon substrates.^[^ ^104^, ^105^ ^]^ The high synthesis temperature ensured the formation of homogeneous alloy structures while simultaneously strengthening the bonding between the HEA/HEO nanoparticles and the carbon substrate, thereby enhancing structural durability. In particular, the smallest possible form of nanoparticles is single‐atom catalysts (SACs), which represent the ultimate downsizing of HEMs to the atomic level, forming single‐atom HEMs. The unique electronic properties and maximum atom utilization make single‐atom HEMs favorable to the catalysis process. For example, a substrate‐mediated SACs formation strategy has been employed to prepare single‐atom HEMs, leveraging reversible redox reactions at the TMDs (substrate)/TM ion interface. The as‐achieved Pt,Ru,Rh,Pd,Re‐MoSe_2_ delivered excellent hydrogen evolution reaction (HER) performance in alkaline conditions.^[^ ^106^ ^]^ Further transitioning from nanoparticles/single atoms to higher‐dimensional structures, such as 1D (nanowires and nanotubes, etc.), 2D (nanosheets, nanoplates, nanospheres, etc.), and 3D (nanoflowers, nanodendrites, hollow structure, etc.), expands the properties and kinetics of HEMs for electrocatalysis.^[^ ^107^, ^108^, ^109^, ^110^, ^111^, ^112^, ^113^ ^]^ For instance, high‐entropy sulfide nanoarrays (CoZnCdCuMnS@CF) were synthesized through a low‐temperature cation exchange reaction by using Co_9_S_8_@CF with a structure of vertical needle‐like nanowires as a precursor, demonstrating exceptional performance for alkaline water splitting (Figure 3d–f).^[^ ^114^ ^]^ A growing variety of 2D HEMs have been developed for water splitting, including sub‐nanometer ribbons, 2D high entropy oxides, and sulfides, layered double hydroxides (LDHs), high entropy metal–organic frameworks.^[^ ^115^, ^116^ ^]^ Moreover, 3D hollow‐structured HEOs (ZnFeNiCuCoRu‐O) feature a large surface area and rapid mass transfer kinetics, delivering exceptional OER catalytic performance across a wide pH range.^[^ ^117^ ^]^

#### 2.2.2 From Random Distribution to Relatively Ordered Structure

HEMs rely heavily on the formation of crystalline or amorphous solid‐solution phases, usually exhibiting a highly disordered atomic arrangement. Specifically, multiple principal elements are randomly distributed within the lattice, leading to increased configurational entropy, which stabilizes the single‐phase solid solution and prevents phase segregation.^[^ ^118^, ^119^ ^]^ The atomic disorder significantly influences the catalytic properties, which are crucial for optimizing catalytic activity and stability. Moreover, the inherent randomness in elemental distribution enables tunable local environments, enhancing synergistic interactions between different elements. For instance, the designed NiFeCoMnAl oxide featured a random distribution of elements, enabling each component to contribute synergistically to OER activity. In particular, Ni served as the primary active site, while the incorporation of Mn created an electron‐rich environment that enhances the catalytic activity of Ni active sites. Co‐doping improved the electrical conductivity, and Al doping facilitates the formation of a nanoporous structure via a dealloying process. This random elemental distribution ensures that each element plays a distinct and complementary role in boosting the overall electrocatalytic efficiency (Figure 3g).^[^ ^120^ ^]^

Although the disordered atomic arrangement of HEMs exhibits unique advantages, this characteristic may potentially hinder the precise control of electronic and catalytic properties. Therefore, understanding how to control the atoms within a chemically ordered distribution and figuring out which atomic configurations benefit the activity remain the challenge in HEM development. Multimetallic alloys or compounds with a particular ordered structure should be promising to overcome this challenge.^[^ ^121^, ^122^, ^123^ ^]^ Local short‐range chemical ordering has been observed in a concentrated solution of single‐phase medium‐entropy alloys (MEA) with a composition near the center of the phase diagram.^[^ ^124^ ^]^ Moreover, the most typical HEMs with relatively ordered structures are high entropy intermetallics (HEIs), which are a class of ordered HEMs where multiple principal elements form an intermetallic compound instead of a random solid solution.^[^ ^125^, ^126^, ^127^, ^128^, ^129^, ^130^ ^]^ HEIs still retain the high configurational entropy while featuring ordered atomic arrangements based on intermetallic compounds, offering unique electronic and catalytic properties. HEIs can be synthesized using parent binary intermetallics, such as PtSn, to serve as the initial reactant. In this structure, Pt sites were partially substituted with Ni and Co, while Sn sites were partially replaced with In and Ga.^[^ ^131^ ^]^ The increased number of constituent metals not only enhances the kinetic stability of the nanoparticles but also suppresses undesired side reactions. The HEIs combine the merits of both high‐entropy and ordered intermetallic properties, potentially enabling specific effects to boost the water‐splitting performance. For instance, the dendrite‐like porous L1_2_‐type HEI, derived from the dual‐phase FeCoNiAlTi HEI (where FeCoNi served as potential active sites and AlTi enhanced the formation of an ordered atomic configuration), exhibited a specific site‐isolation effect that further stabilizes H_2_O/H* adsorption and desorption (Figure 3h).^[^ ^43^ ^]^ This significantly optimizes the energy barrier for hydrogen evolution.

#### 2.2.3 From Bare HEMs to Surface Reconstructed HEMs

**Figure 4:**
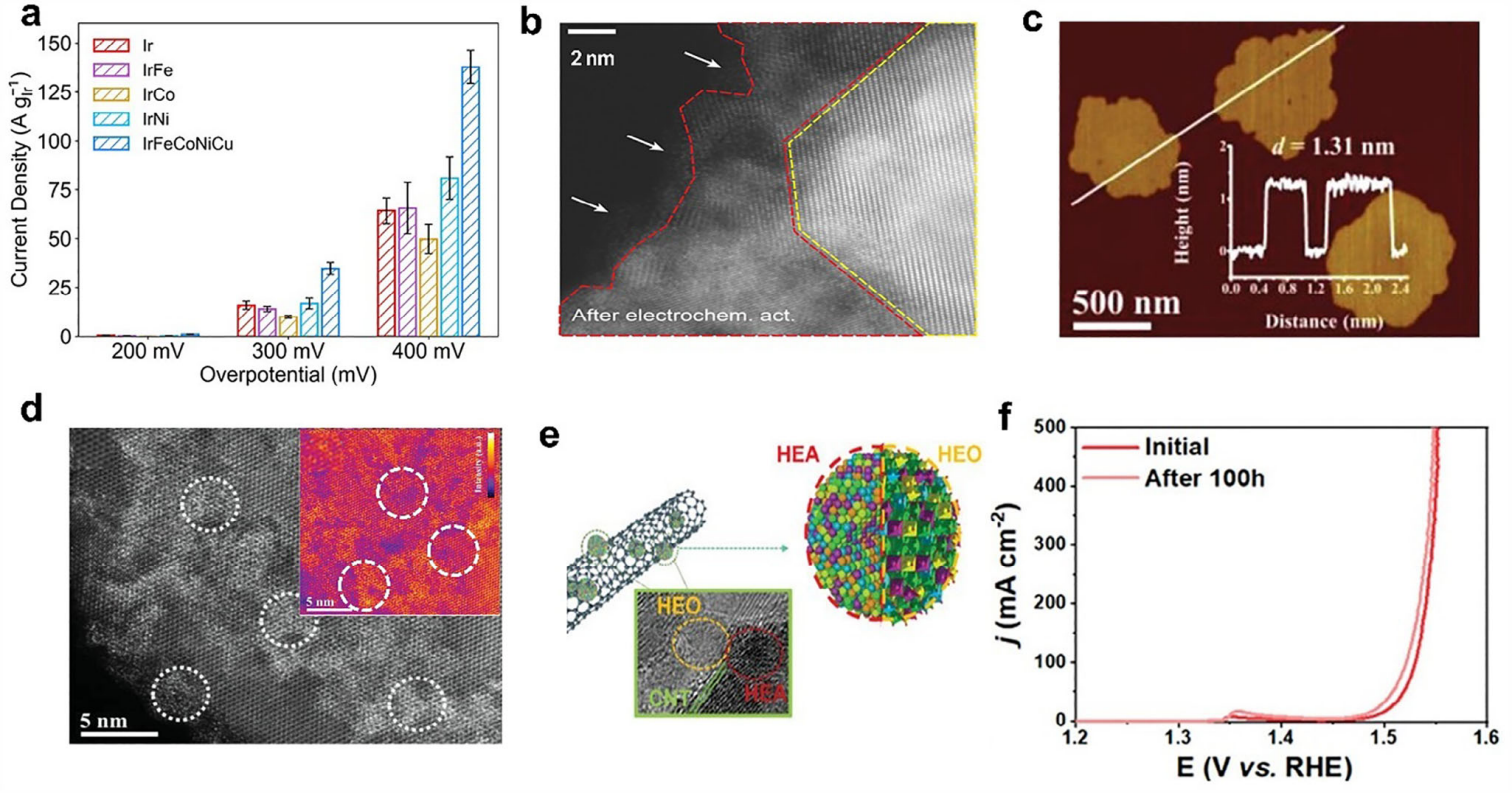
a) The HER activity comparison of IrFeCoNiCu HEA and other Ir‐based catalysts at different overpotentials. b) High resolution HAADF‐STEM image showing an Ir‐enriched surface layer (indicated by white arrows) on the HEA core after electrochemical activation. a,b) Reproduced under the terms of the CC‐BY Creative Commons Attribution 4.0 International license.^[^ ^132^ ^]^ Copyright 2023, American Chemical Society. c) AFM image and corresponding height profile of the HEA‐PdPtRhIrCu metallene. d) HAADF‐STEM image and false‐color aberration‐corrected image of the HEA‐PdPtRhIrCu metallene. c,d) Reproduced with permission.^[^ ^142^ ^]^ Copyright 2023, Wiley‐VCH. e) Diagram of FeCoNiMnCr HEA‐HEO/CNT catalyst. f) Polarization curves before and after the stability test. e,f) Reproduced with permission.^[^ ^149^ ^]^ Copyright 2024, Wiley‐VCH.

Further engineering HEMs to form appealing structures by refining their structural, electronic, and surface properties can induce additional enhancements, leading to improved catalytic efficiency and stability for water splitting. For instance, selective leaching of 3d transition metals during the electrochemical activation process leads to the formation of a precious metal‐rich surface. A typical example is IrFeCoNiCu HEA, which resulted in an Ir‐enriched surface layer that significantly enhanced OER catalytic capability for a low overpotential of ≈302 mV at 10 mA cm^−2^ (**Figure** **4**a,b).^[^ ^132^ ^]^ Surface reconstruction to form an oxidized or reduced layer on the surface of HEMs is another emerging strategy to dynamically change the atomic arrangement and electronic structure, leading to unconventional properties.^[^ ^133^, ^134^, ^135^ ^]^ Especially, the non‐HEOs (e.g., HEAs or HE metal compounds) underwent rapid in situ electrochemical oxidation, into metal (oxy)hydroxides or oxides, which are considered the real OER active sites in alkaline solution.^[^ ^136^, ^137^, ^138^, ^139^ ^]^ For instance, the surface states of HEA sheets (Ti, Cr, Mn, Fe, Co, Ni, Zr, Nb, and Mo) exhibited no significant changes between the OCP and HER state, indicating that the metallic nature of the HEA was maintained during the reduction processes.^[^ ^140^ ^]^ In contrast, during the OER process, all elements in the HEAs were partially oxidized to form oxide layers. Similarly, FeNiCoCrMnS_2_ also experienced in situ structural self‐reconstruction during OER, fully converting into metal (oxy)hydroxide at 1.2 V versus RHE, which served as the real active sites for OER. Additionally, the remaining sulfate anion also synergistically boosted the OER catalytic activity.^[^ ^141^ ^]^

#### 2.2.4 From Intact HEMs to Defective HEMs

Other than surface reconstruction, forming defective HEMs can also significantly influence their physicochemical properties, which directly affect the adsorption of the reactants/intermediates. There are various defects, such as lattice distortion, stacking faults, and vacancies, observed in HEMs, which can be formed during the preparation process.^[^ ^142^, ^143^ ^]^ For instance, an ultrathin HEA‐PdPtRhIrCu metallene rich in lattice distortions and defects was synthesized through a simple hydrothermal method (Figure 4c,d).^[^ ^142^ ^]^ While atomic‐level mixing of multiple elements may already be effective to form defects, developing methods to specifically introduce desired defects for HEMs is more significant. Plasma strategy, electrochemical dealloying, high‐temperature treatment, etc., have been applied to create cation and anion vacancies for HEMs. For example, low‐temperature plasma techniques facilitated the transformation of LDHs into HEO nanosheets with abundant oxygen vacancies and a high surface area.^[^ ^144^ ^]^ A low‐temperature surface carbonization–decarbonization strategy has also been developed by encapsulating high‐entropy oxides with a carbonaceous layer and precisely controlling the decomposition to introduce and modulate oxygen vacancies in CoMnFeNiZn‐O_v_ HEOs.^[^ ^143^ ^]^ The oxygen vacancies formed in HEOs enhance both *OH adsorption and deprotonation simultaneously, leading to excellent OER activity.

#### 2.2.5 From Pristine HEMs to Functionalized HEMs

Similar to conventional catalysts, the surface of pristine HEMs can be functionalized by introducing additional foreign species, enabling appealing properties and higher catalytic activity. Non‐metal doping (e.g., N, F, P, and B) is an effective strategy for tailoring the properties of HEMs. For example, N incorporation into NFeCoNiAlMoO_x_ activated lattice oxygen by modulating the hybridization between active site Mo 3d orbitals and O 2p orbitals, thereby enhancing OER activity.^[^ ^145^ ^]^ Another effective and straightforward approach is the incorporation of single atoms, clusters, or nanoparticles, which introduces cocktail effects to enhance the reaction kinetics and durability of HEMs. Alloying Pd with the surface atoms on non‐noble HEA‐NPs enhanced catalyst stability while minimizing noble metal usage.^[^ ^146^ ^]^ Ag clusters incorporated in high entropy CuCoFeAgMo (oxy)hydroxides could efficiently lower the limiting energy barrier, facilitating improved reaction kinetics, while their higher Fermi levels served as electron donors to activate the metal sites.^[^ ^147^ ^]^ This cocktail effect optimizes charge transfer and adsorption energy, ultimately leading to superior OER performance. Beyond metal elements, the surface of HEMs can also be functionalized with non‐metallic components or stabilized on carbon substrates. Stabilizing HEMs on conductive carbon‐based substrates can significantly improve electrical conductivity, structural integrity, and mechanical stability, ensuring long‐term stability for water splitting. A typical example is CuNiFeCoCrTi@N‐doped graphene nanoparticles (CuNiFeCoCrTi@NC NPs) with precisely controlled N‐doped graphene shell layers.^[^ ^148^ ^]^ Reducing the graphene layer thickness and increasing the number of elements in the alloy significantly boosted the HER performance. Moreover, constructing high‐entropy heterostructures can enhance structural stability and corrosion resistance, contributing to sustained OER activity under harsh electrochemical conditions. A Cr‐induced spontaneous reconstruction strategy was employed to synthesize FeCoNiMnCr HEA and HEO heterocatalysts.^[^ ^149^ ^]^ These materials provide active sites with stable valence states, ensuring long‐term durability for OER, even under industrial operating conditions (Figure 4e,f).

## 3 Elemental Roles in High Entropy Materials

**Figure 5:**
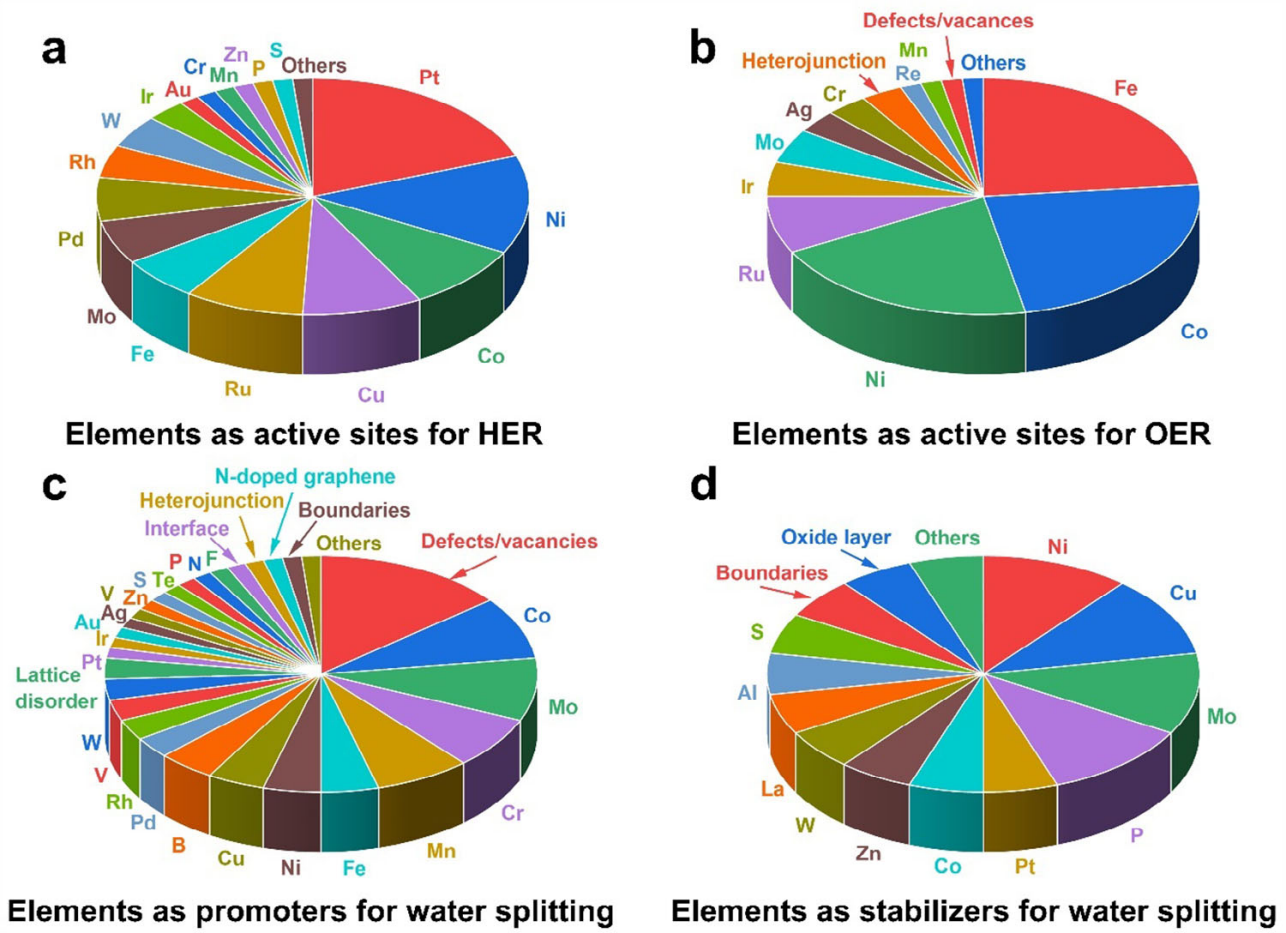
The element role of HEMs as (a,b) active sites, c) promoters and (d) stabilizers for water splitting.

The diverse elemental composition of HEMs endows them with a range of unique synergistic properties, making them highly promising for water splitting.^[^ ^150^, ^151^ ^]^ However, this same complexity also presents a significant challenge to understanding the individual role of each element. In HEMs, each element can contribute differently, such as serving as active sites, increasing conductivity, maintaining corrosion resistance, and promoting stability. Therefore, clearly understanding the influence of each element on the overall structure, stability, and functional performance of HEMs is critical for the rational design and optimization of these HEMs. It is well established that the catalytic performance of water splitting (HER and OER) is closely related to the adsorption energy of reactants or intermediates at active sites of HEMs. Achieving optimal catalytic activity typically requires an optimized adsorption strength, which is often determined by the active sites. Thus, identifying the active sites of HEMs for water splitting is considered the first vital approach. By tuning the properties or electronic structure of HEMs through the structural evolution discussed in Section 2, the adsorption energy can be precisely modulated to enhance catalytic performance in water splitting. This highlights the importance of identifying not only the elements that serve directly as active sites but also those that, while not directly involved in catalysis, still play significant roles in influencing overall catalytic activity. In addition to catalytic activity, the stability of HEMs is a critical factor influencing overall catalytic performance. Understanding how individual elements contribute to stability is also essential. This session will focus on exploring the atomic‐level roles of individual elements within HEMs, aiming to uncover fundamental insights that drive material innovation. We will examine the elements in terms of three distinct roles: active sites, promoters, and stabilizers (**Figure** **5**).

A few approaches have been applied to can identify the element role of HEMs for water splitting. The first approach is the poisoning experiment. The roles of metal species are typically identified using poisoning agents that selectively bind to specific metals, reducing catalytic activity and indicating the functional sites.^[^ ^152^, ^153^, ^154^ ^]^ However, HEMs generally contain five or more metal elements, and many show similar properties, which makes it challenging to pinpoint the element roles using this approach. In situ characterization techniques are widely employed to study the water splitting process under realistic operating conditions, offering valuable insights into the underlying relationship between catalyst structure and active sites. For instance, operando IR and Raman spectroscopy are particularly important for characterizing HEMs, as they enable direct monitoring of interactions between intermediates and the catalyst surface (binding sites). Additionally, in situ, X‐ray absorption spectroscopy allows the monitoring of the dynamic process of water splitting, revealing changes in electronic states, identifying active sites, and uncovering synergistic effects within HEM catalysts.^[^ ^125^, ^155^, ^156^ ^]^ Meanwhile, due to the complexity of HEMs, DFT calculations play essential roles in identifying the element roles for water splitting.^[^ ^157^ ^]^ As the HER or OER catalytic activity is largely governed by the inherent electronic structure of HEMs, the d‐band theory is developed to predict the bonding interactions between HEM surfaces and intermediates. The total density of states (DOS) is commonly used to indicate the conductivity of HEMs, with a higher DOS near the Fermi level facilitating more efficient electron transfer. The projected density of states (PDOS), derived from the total DOS, provides detailed insights into the electronic structure and is strongly correlated with HER and OER activity, which is a good indicator to identify the element roles. ΔG diagrams are widely recognized as a powerful tool for identifying the rate‐determining step (RDS) in the HER and OER pathways. The activity of the active sites directly influences the adsorption energy of reactants or intermediates, as adsorption onto these sites is a necessary first step to initiate the catalytic process.^[^ ^158^, ^159^, ^160^ ^]^

### 3.1 Elements as Active Sites

**Figure 6:**
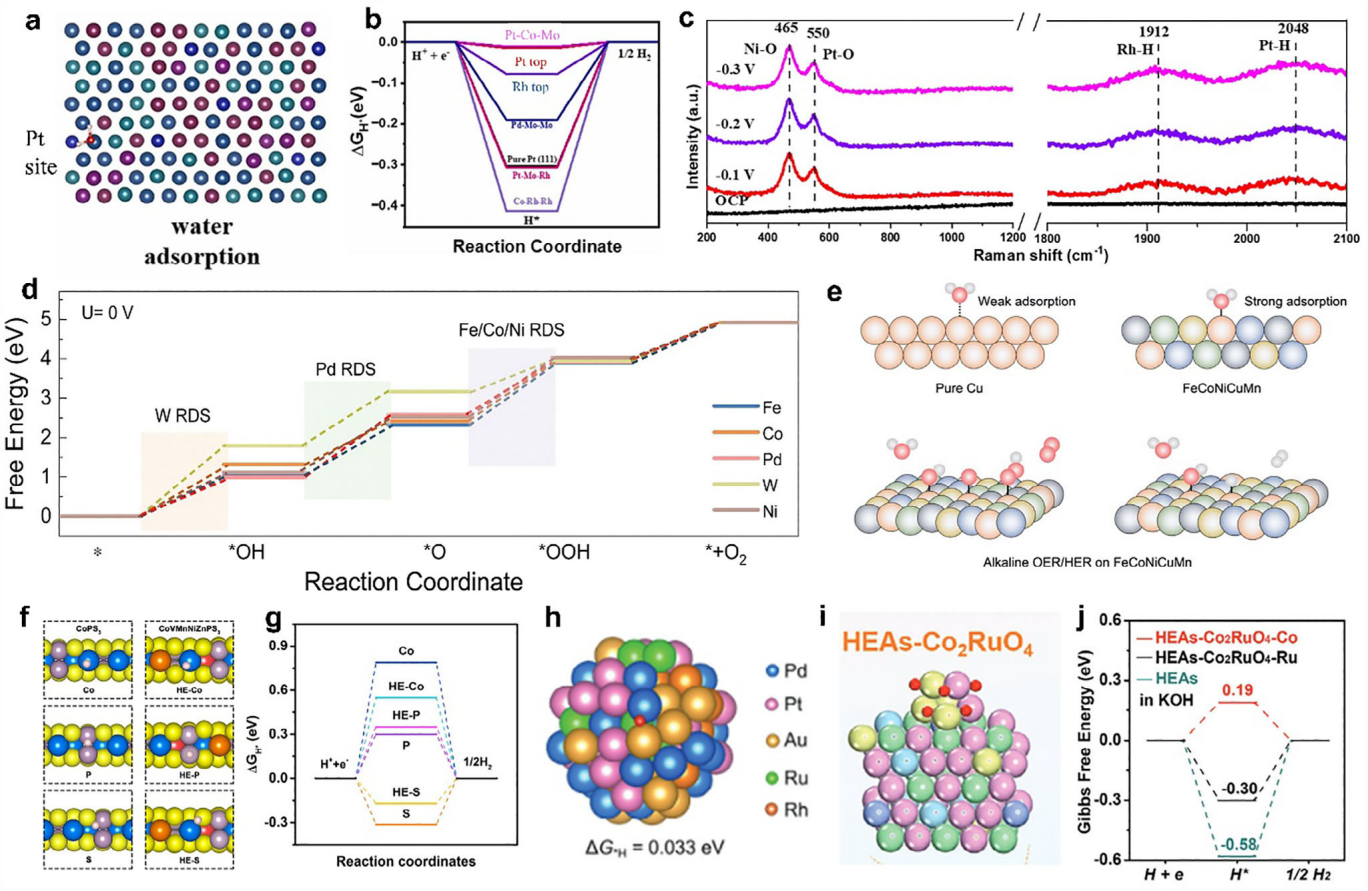
a) Binding configurations and b) Adsorption free‐energy landscape of atomic hydrogen on of Pt_30_Mo_5_Pd_25_Rh_25_Ni_15_(111). a,b) Reproduced with permission.^[^ ^58^ ^]^ Copyright 2023, Elsevier. c) In situ SERS with different applied potentials of Pt_28_Mo_6_Pd_28_Rh_27_Ni_15_NCs. c) Reproduced with permission.^[^ ^166^ ^]^ Copyright 2023, Royal Society of Chemistry. d) Standard free energy diagram of the OER process at 0 V of FeCoNiPdWOOH for various active sites. d) Reproduced with permission.^[^ ^172^ ^]^ Copyright 2024, Royal Society of Chemistry. e) Scheme of the different adsorption strengths of intermediates on metal sites in HEAs and Cu catalysts and reaction pathways of the alkaline HER and OER at the Cu sites in the HEA NPs. e) Reproduced with permission.^[^ ^110^ ^]^ Copyright 2023, Royal Society of Chemistry. f) Edge models of Co, P, and S sites for CoVMnNiZnPS_3_ and CoPS_3_. g) Gibbs free‐energy profile of HER occurring on the characterized edge configurations (f). f,g) Reproduced under the terms of the CC‐BY Creative Commons Attribution 4.0 International license.^[^ ^88^ ^]^ Copyright 2022, American Chemical Society. h) Adsorption sites of Pd‐Au bridging on the surface of HEA‐1.4 and adsorption energies of hydrogen atoms. h) Reproduced with permission.^[^ ^176^ ^]^ Copyright 2025, Wiley‐VCH. i) Optimal adsorption intermediates and j) Adsorption free‐energy on HEAs‐Co_2_RuO_4_‐Ru. i,j) Reproduced with permission.^[^ ^181^ ^]^ Copyright 2025, Wiley‐VCH.

HEMs exhibit a complex composition of multiple metal species, resulting in a diverse array of potential active sites for HER and OER. In particular, precious metals such as Pt, Pd, Ir, and Ru in HEMs have demonstrated their capability to significantly enhance catalytic activity. In Pt‐based HEMs, Pt atoms typically serve as the primary active sites for HER, as observed in compositions such as PtIrFeCoNi, PtPdRuIrAu, and PtCoMoPdRh.^[^ ^161^, ^162^, ^163^ ^]^ For instance, the investigation for PtCoMoPdRh indicates that the ΔG_H*_ values at most active sites were superior to that of the Pt(111) surface, highlighting the significant support from the high entropy elements to the active sites within the HEA (**Figure** **6**a,b).^[^ ^58^ ^]^ Especially, hydrogen adsorption on Pt top sites forms stable M─H* bonds with the lowest ΔG_H*_, confirming that these Pt top sites served as the primary and most active centers during the Tafel step of the HER mechanism. Similar to Pt‐based HEMs for HER, in Ir‐based HEMs including IrFeCoNiCu HEA nanoparticles and Ir‐rich IrRuNiMo shell@IrRuCoNiMo core structures,^[^ ^53^, ^164^ ^]^ Ir atoms typically act as the primary active sites for OER. These catalysts exhibit enhanced OER activity compared to monometallic Ir and other highly active Ir‐based bimetallic alloys. Other precious metals in HEMs, such as Ru in FeCoNiMoPtRu, Rh in PtRhNiFeCu and PtMoPdRhNi, and Pd in PtPdIrRuAu can act as active sites for HER or OER, either independently or collaboratively boost the performance.^[^ ^86^, ^165^, ^166^, ^167^ ^]^ For example, in situ Raman spectroscopy has identified characteristic peaks at 1912 and 2048 cm^−1^, corresponding to Rh─H and Pt─H bonds, respectively. These findings confirm that Pt and Rh served as the primary active sites for H* adsorption and desorption in Pt_28_Mo_6_Pd_28_Rh_27_Ni_15_ NCs (Figure 6c).^[^ ^166^ ^]^

Moreover, both catalytically active transition metals (such as Fe, Co, and Ni) and typically inactive ones (such as Cu and Zn) can serve as effective active sites in HEMs for HER and OER. This is attributed to the unique high‐entropy structures that create a highly disordered atomic environment to modulate the local electronic structure and facilitate synergistic interactions among multiple elements, which may enhance the catalytic capability these metal species as active sites for HER and OER. For example, the active transition metals Ni in NNM‐HEA@CF, Ni in FeCoNiMoW HEA, Fe/Ni in CoFeNiCrMnP/NF, Fe/Co/Ni in CrMnFeCoNi, Fe/Co/Ni in (FeNiCoCrMnV) HEO, etc. have demonstrated catalytic contributions to the HER or OER process.^[^ ^36^, ^67^, ^168^, ^169^, ^170^, ^171^, ^172^ ^]^ Specifically, FeCoNiPdWP high‐entropy phosphide (HEP) nanoparticles undergo surface reconstruction to form a FeCoNiPdW high‐entropy oxyhydroxide, in which Fe, Co, and Ni with high oxidation states act as active sites for OER. As a result, this catalyst exhibits an exceptionally low OER overpotential of 227 mV at a current density of 10 mA cm^−2^ (Figure 6d). In addition to active transition metals, traditionally inactive metals in HEMs can also function as catalytic active sites for HER and OER. This unexpected activity arises from the unique local chemical environments and electronic structure modulation induced by the high‐entropy configuration, which can activate inert metals by altering their adsorption energies. For instance, the electronegativity differences among Mn, Cu, Fe, Co, and Ni induce local electron redistribution, increasing electron density at the Ni and Cu sites. (Figure 6e) This electron enrichment activated Cu sites to serve as effective active sites for both HER and OER. Therefore, the FeCoNiCuMn HEA nanoparticles demonstrated excellent electrocatalytic performance, achieving an overpotential of 281 mV at 100 mA cm^−2^ for HER and 386 mV at 200 mA cm^−2^ for OER.^[^ ^110^ ^]^ Moreover, in the H‐FeCoNiCuMo or Fe FeNiCuWRu catalyst, the inactive Cu could collaborate with Co or Ni to promote the HER process, resulting in exceptional electrocatalytic activity and long‐term stability.^[^ ^173^ ^]^ In addition to metal sites, non‐metallic sites can also be identified as active centers. For example, in Co_0.6_(VMnNiZn)_0.4_PS_3_ nanosheets, Mn sites play a crucial role in facilitating water dissociation. Simultaneously, optimized sulfur sites at the edges and phosphorus sites on the basal plane serve as active sites for hydrogen adsorption (Figure 6f,g).^[^ ^88^ ^]^ This synergistically enhances HER performance, achieving a low overpotential of 65.9 mV at 10 mA cm^−2^.

Beyond the commonly recognized top sites, bridge and hollow sites can also serve as effective active sites to enhance HER or OER performance.^[^ ^50^, ^86^, ^163^, ^174^, ^175^ ^]^ For example, Pd‐Au bridge sites have been shown to contribute significantly to HER activity (Figure 6h).^[^ ^176^ ^]^ Among various PdPtRuRhAu HEAs with different particle sizes, the sample with an average size of 3.14 nm exhibited the highest proportion of bridge sites (18.97%), resulting in the best catalytic performance and achieving an overpotential of just 70.07 mV at a current density of 10 mA cm^−2^. Similarly, in the (RuSnSbReF)O_x_ electrocatalyst, Ru–Re bridge sites enabled electron transfer from Ru to Re via oxygen bridges, thereby tuning the electronic structure and oxidation states.^[^ ^177^ ^]^ These bridge sites served as active centers for acidic OER, playing a key role in determining the energy barrier of the RDS. Hollow sites have also been identified as active centers, offering additional adsorption configurations that further promote reaction kinetics. In the PtNiFeCoCu HEA, Fe sites actively promote water adsorption, initiating the alkaline HER process.^[^ ^44^ ^]^ The resulting OH species were stabilized by adjacent hollow sites, while hydrogen adsorption preferentially occurred at hollow sites near Ni and Co atoms, facilitating efficient HER activity under alkaline conditions.

Reconstructed surface species can also serve as active sites for water splitting.^[^ ^178^, ^179^, ^180^ ^]^ Ag‐decorated Co‐Cu‐Fe‐Ag‐Mo (oxy)hydroxide (Ag@CoCuFeAgMoOOH) electrocatalysts were synthesized via an electrochemical reconstruction method.^[^ ^147^ ^]^ Remarkably, the Ag sites within the Ag clusters acted as comparably active sites for OER, synergistically enhancing the catalytic performance by achieving a low overpotential of 270 mV at 100 mA cm^−2^. Moreover, the optimized self‐reconstructed PtRu_2.9_Fe_0.15_Co_1.5_Ni_1.3_HEA, featuring an interface with cobalt ruthenate (HEAs‐Co_2_RuO_4_), demonstrated enhanced HER catalytic activity (Figure 6i,j).^[^ ^181^ ^]^ In this heterostructure, Co^2+^ and Ru^3+^ species in Co_2_RuO_4_ functioned as active sites for both H_2_O dissociation and hydrogen adsorption, which is different from pure HEA, where Ru atoms within the alloy were the main active sites. This synergistic interface significantly boosted alkaline HER performance, with HEAs‐Co_2_RuO_4_ achieving a current density of 41.8 mA cm^−2^ at 0.07 V versus RHE, which is 2 times higher than PtRu_2.9_Fe_0.15_Co_1.5_Ni_1.3_ HEA.

### 3.2 Elements as Promoters

**Figure 7:**
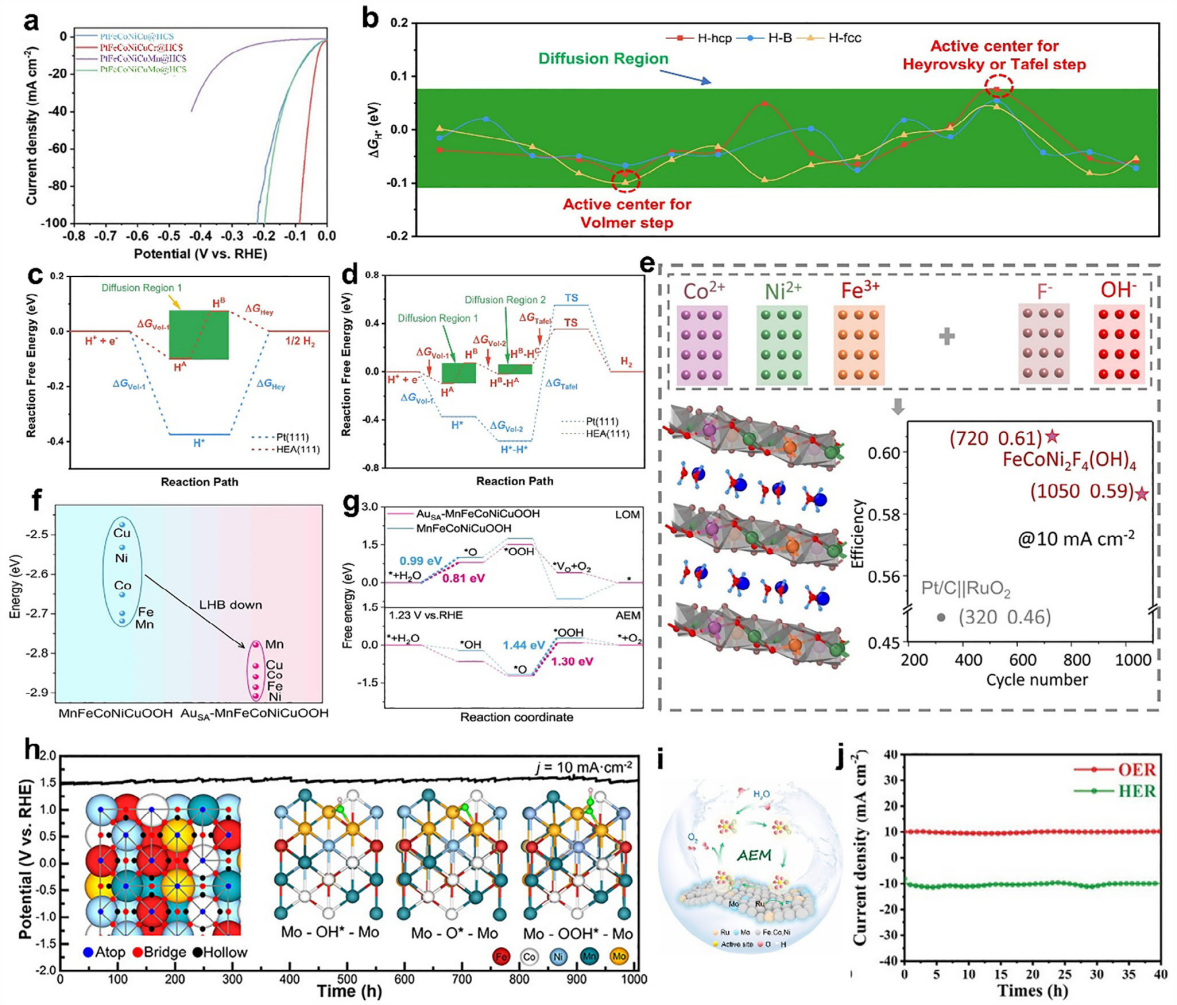
a) HER polarization curves of PtFeCoNiCuCr@HCS. a) Reproduced with permission.^[^ ^184^ ^]^ Copyright 2024, Wiley‐VCH. b) Δ*G*_H*_ distribution on 5.9%‐HEA (5.9% compressive strain) showing adsorption sites (hcp, fcc, bridge). Red dashes mark Volmer/Heyrovsky (or Tafel) active sites; green indicates H* diffusion (DR). c) Volmer–Heyrovsky mechanism and d) Volmer–Tafel mechanism of HER on 5.9%‐HEA (111) and Pt (111). b–d) Reproduced under the terms of the CC‐BY Creative Commons Attribution 4.0 International license.^[^ ^185^ ^]^ Copyright 2024, Springer Nature. e) Active site model of HEA LDH and its electrochemical properties. e) Reproduced with permission.^[^ ^186^ ^]^ Copyright 2024, Wiley‐VCH. f) The LHB center positions and (g) Computed free energies (Δ*G*) of OER steps of Au_SA_‐MnFeCoNiCuOOH and MnFeCoNiCuOOH. f,g) Reproduced under the terms of the CC‐BY Creative Commons Attribution 4.0 International license.^[^ ^24^ ^]^ Copyright 2023, Springer Nature. h) ToF‐SIMS image of the FeCoNiMnMo SPHEA after 1000 h of continuous stability testing. h) Reproduced under the terms of the CC‐BY Creative Commons Attribution 4.0 International license.^[^ ^195^ ^]^ Copyright 2022, American Chemical Society. i) Active site model of HE‐MOFs. i) Reproduced under the terms of the CC‐BY Creative Commons Attribution 4.0 International license.^[^ ^196^ ^]^ Copyright 2024, American Chemical Society. j) current density–time curve of FeCoNiRu‐450. j) Reproduced under the terms of the CC‐BY Creative Commons Attribution 4.0 International license.^[^ ^197^ ^]^ Copyright 2022, Wiley‐VCH.

While some elements in HEMs may not act as direct active sites for water splitting, they can play a crucial role as promoters by modulating the geometric configuration and electronic structure of neighboring active sites. These promoter elements can influence factors such as electron density, oxidation states, adsorption energy, and coordination environment, thereby enhancing the intrinsic catalytic activity of the true active sites. Their presence contributes to the synergistic effect which is often observed in HEMs, enabling fine‐tuning of surface properties and improved overall catalytic performance. One of the most common mechanisms for the rest elements as promoters to enhance catalytic performance in HEMs is through electron transfer between the promoter elements and the active sites due to their close interaction.^[^ ^26^, ^182^, ^183^ ^]^ For instance, in the PtFeCoNiCuCr@HCS, Cr primarily acted as an electron donor, modulating the electronic configuration of the surrounding active sites to enhance their catalytic properties (**Figure** **7**a).^[^ ^184^ ^]^ Co served as the principal active site for water dissociation to generate hydrogen intermediates, which preferentially migrated toward Pt, Fe, and Cu sites, where hydrogen molecule formation occurred. In addition, the size mismatch between different elements and active sites in HEAs can induce compressive strain on the catalyst surface, which in turn enhances catalytic activity. For example, in the PtFeCoNiCu HEA, the atomic radii of Fe, Co, Ni, and Cu are all smaller than that of Pt.^[^ ^185^ ^]^ When these smaller atoms were incorporated into the HEA matrix, they exerted compressive strain on the surrounding Pt atoms at the surface. This strain effect modified the local electronic structure of the Pt active sites, resulting in an optimized H* adsorption on surface Pt sites, which promotes the HER process (Figure 7b–d). Non‐metal can also work as promoters, including N, P, F, Te, and B, to facilitate the enhancement of the OER process. F species increased the overlap between Co/Fe 3d and O 2p energy bands, thereby enhancing the covalency of Co─O and Fe─O bonds, which promoted more efficient electron transfer between Co/Fe and the OER intermediates, contributing to the high intrinsic catalytic activity of γ‐FeCoNi_2_F_4_(OH)_4_ (Figure 7e).^[^ ^186^ ^]^ The incorporation of non‐metallic Te in CoFeNiMoWTe N‐HECGs facilitated efficient electron transfer between Te and metal species, leading to increased valence states of metal cations that promote OER activity.^[^ ^187^ ^]^ Additionally, nitrogen (N), another non‐metallic element, activated lattice oxygen by modulating the hybridization between Mo 3d orbitals (active sites) and O 2p orbitals in NFeCoNiAlMoO_x_.^[^ ^145^ ^]^ This modification enables a shift in the reaction pathway from the adsorbate evolution mechanism (AEM) to the lattice oxygen mechanism (LOM) during the formation of *OOH in step 3. The synergistic effect of AEM and LOM significantly boosts OER activity.

Defects or vacancies can act as promoters to enhance the catalytic performance of HEMs.^[^ ^143^, ^188^ ^]^ In (MoWVNbTa)C, W, and Mo serve as the most active sites for the HER.^[^ ^100^ ^]^ Further investigations reveal that these active sites, when adjacent to vacancies, exhibit more favorable Δ*G*_H*_ values than those in a defect‐free structure. This improvement is attributed to the shielding effect of vacancies on electron transfer, leading to optimized Δ*G*_H*_ and high HER activity. Similarly, the presence of phosphorus vacancies (*V*_P_) can effectively activate the surrounding Co atoms, which are the active sites in V_P_‐CoNiCuZnFeP.^[^ ^189^ ^]^ Twin boundaries, as planar defects, also play a key role in modulating surface electronic structures. When combined with the promoter effect from electron‐rich B atoms in B‐doped FeCoNiCuMoB HEA film, they lead to optimized atomic configurations that synergistically promote a favorable pathway for both HER and OER.^[^ ^116^ ^]^

The incorporated species or forming heterostructure can also act as promoters, modifying the electronic structure of the active sites. In V_x_CuCoNiFeMn, the incorporation of V alters the electronic environment of neighboring elements (particularly Cu and Fe) through electron sharing from the high‐valent V.^[^ ^190^ ^]^ This electronic modulation shifts the preferred active site for H_2_O adsorption and dissociation from Fe (in the pristine CuCoNiFeMn) to Cu sites (in V_x_CuCoNiFeMn). The resulting H^+^ is then readily adsorbed on adjacent Fe sites, where the binding energy barrier is reduced due to the optimized electronic structure of V_1.0_‐HEA, facilitated by efficient electron transfer. As a result, the engineered V_x_CuCoNiFeMn exhibits an overpotential of 250 mV at 50 mA cm^−2^, which is ≈170 mV lower than that of non‐engineered HEA (422 mV). Incorporating Au single atoms into MnFeCoNiCu (Au_SA_‐MnFeCoNiCu LDH) promotes the release of lattice oxygen, thereby favoring the LOM and enhancing the intrinsic catalytic activity (Figure 7f,g).^[^ ^24^ ^]^ Au_SA_‐MnFeCoNiCu LDH achieves a low overpotential of 213 mV at 10 mA cm^−2^ and an impressive mass activity of 732.925 A g^−1^ at an overpotential of 250 mV for alkaline OER. Carbon materials, including carbon nanotubes (CNTs) and graphene, can also act as effective promoters for HEMs in water splitting. The hybridization between the metal d‐orbitals of the RuPdIrPtAu HEA and the π‐electrons of CNTs induces localized charge redistribution at the interface between CNTs and HEA, highly facilitating charge transfer and electronic coupling.^[^ ^191^ ^]^ This synergy enables ultralow overpotentials of 30.7 mV for HER and 330 mV for OER at a current density of 10 mA cm^−2^. Moreover, the heterostructure facilitates electron transfer, thereby enhancing the intrinsic catalytic activity of the active sites. The engineered core–shell FeCoNiMoAl‐based HEA features a shell that lowers electron transfer resistance and offers supplementary active sites, with a crystalline core that supports improved conductivity and long‐term stability.^[^ ^192^ ^]^ Moreover, charge redistribution at the core–shell interface elevates the valence states of Ni and Co species, further promoting the OER process.

### 3.3 Elements as Stabilizers

One disadvantage of HEMs is their sluggish diffusion effect on kinetics, which significantly influences the phase transitions. The presence of multiple elements with varying atomic radii and chemical activities in HEMs leads to lattice distortion, which impedes atomic movement and limits diffusion rates.^[^ ^193^, ^194^ ^]^ The suppressed diffusion impacts not only phase stability but also plays a pivotal role in governing the overall performance of the HEM system. For instance, the as‐prepared FeCoNiMnMo HEMs demonstrate excellent electrocatalytic performance for OER, achieving a low overpotential of 279 mV at 10 mA cm^−2^ and maintaining remarkable stability for over 1000 h.^[^ ^195^ ^]^ The results reveal Mo as a key contributor to the enhanced catalytic activity and long‐term durability. The high‐entropy effect plays a vital role in suppressing Mo leaching, thereby preserving the structural integrity and thickness of the active catalytic layer throughout the OER process (Figure 7h). Moreover, the incorporation of high‐valence metals such as Mo and W into CoFe‐based oxides impart self‐healing properties to the electrocatalysts, enabling them to maintain high OER activity and long‐term stability.^[^ ^90^ ^]^

In addition to the intrinsic high‐entropy effect, which provides enhanced structural and chemical stability compared to single‐metal electrocatalysts, many HEMs are further engineered through the incorporation of specific stabilizing elements. These added elements can improve resistance to corrosion, suppress the dissolution of active sites under harsh electrochemical conditions, and maintain the integrity of the active phase during long‐term water‐splitting operations. The atomic‐level dispersion of Ru and Mo within HE(Ru,Mo)‐MOF nanosheets leads to the formation of densely packed O‐bridged RuMo dual‐atom active sites.^[^ ^196^ ^]^ The incorporation of Mo promotes the stabilization of Fe and Ni in lower oxidation states (+2), contrasting with the effect of Ru, which tends to induce higher oxidation states of Fe and Ni. Additionally, in Mo‐doped HE(Ru,Mo)‐MOF samples, the Ru 3p peaks shift toward higher binding energies, indicating a decrease in electron density around Ru atoms due to electronic interactions with Mo. These findings suggest that the high‐valent Mo species help balance and stabilize the overall coordination environment and electronic structure, thereby enhancing the long‐term stability of HE(Ru,Mo)‐MOFs for OER (Figure 7i).

The formation of surface layers plays a critical role in enhancing the structural and electrochemical stability of HEMs during water splitting. The surface layers enriched with specific elements can act as protective barriers against corrosion and dissolution under harsh electrochemical conditions. Stability studies on the FeCoNiRu HEA reveal that its exceptional durability is primarily attributed to the formation of a spinel oxide surface layer during the electrochemical reaction.^[^ ^107^ ^]^ This spinel structure effectively preserves the active sites derived from the intrinsic architecture of the HEA, thereby maintaining high catalytic performance and contributing to the material's remarkable stability (Figure 7j). Moreover, the oxidized P and S species in high‐entropy phosphorus sulfide (HEPS) form an anionic protective layer that shields the catalytically active metal sites from aggregation and dissolution.^[^ ^197^ ^]^ Simultaneously, the in situ formation of V_2_O_x_ species serves as a stabilizer to against additional oxidation of HEPS, further enhancing the durability for OER. As a result, maintains stable performance for over 1200 h in a practical electrolyzer with negligible activity loss.

## 4 High Entropy Materials for Water Splitting

Water splitting is a promising method to produce green hydrogen. However, efficient water splitting requires catalysts that can sustain high catalytic activity and stability under extreme operational conditions (acidic and alkaline media). HEMs, composed of multiple principal elements with altered electronic structures, surface chemistries, adsorption properties, and stability, can significantly boost the catalytic performance for HER and OER. Moreover, HEMs offer the flexibility to incorporate more abundant and cost‐effective elements into the precious metals (Pt, Ir)‐based electrocatalysts to reduce the cost with no compromise of the catalytic performance. This section will focus on HEMs for water splitting, highlighting their unique properties and the specific roles of each element (**Table** **1** and **2**).

### 4.1 Hydrogen Evolution Reaction

The primary strategy for generating hydrogen through electrocatalysis is HER, a key half‐reaction in water splitting. HER involves two‐electron transfer with multiple reaction steps occurred at the cathode–electrolyte interface. The proton source for HER depends on the electrolyte pH, that is, H_2_ produced via the reduction of H_3_O^+^ in acidic conditions and the reduction of H_2_O in alkaline media. There are two steps for HER in acidic conditions, including the Volmer reaction with the adsorption of hydrogen atoms on the catalyst surface (forming H*), and the Heyrovsky or the Tafel reaction when H_2_ is produced. In the Heyrovsky pathway, a hydrogen molecule is formed by the combination of an adsorbed hydrogen atom (H*) and the proton (H^+^) from the electrolyte with one electron from the electrode. In contrast, the Tafel reaction involves the direct combination of two adjacent adsorbed hydrogen atoms (H*) to produce molecular hydrogen (H_2_). Similarly, HER in alkaline conditions also proceeds via either the Volmer–Heyrovsky or Volmer–Tafel mechanisms. However, an additional water dissociation step is required to generate H* and OH^−^ (Volmer step). Subsequently, H* reacts with another water molecule and an electron to form H_2_, a step known as the Heyrovsky step in alkaline conditions. Alternatively, the Tafel step, involving the combination of two H* species to form H_2_, remains unchanged from that in acidic media. The additional water dissociation step introduces an extra energy barrier and leads to a higher overpotential for HER in alkaline conditions. Therefore, highly efficient electrocatalysts are essential for reducing the overpotential and improving the catalytic performance of HER. Among various catalysts, HEMs have emerged as promising candidates for HER due to their unique properties, including the high‐entropy effect, lattice distortion effect, and cocktail effect.^[^ ^198^, ^199^, ^200^ ^]^

#### 4.1.1 Pristine Noble Metal‐Based HEMs

**Figure 8:**
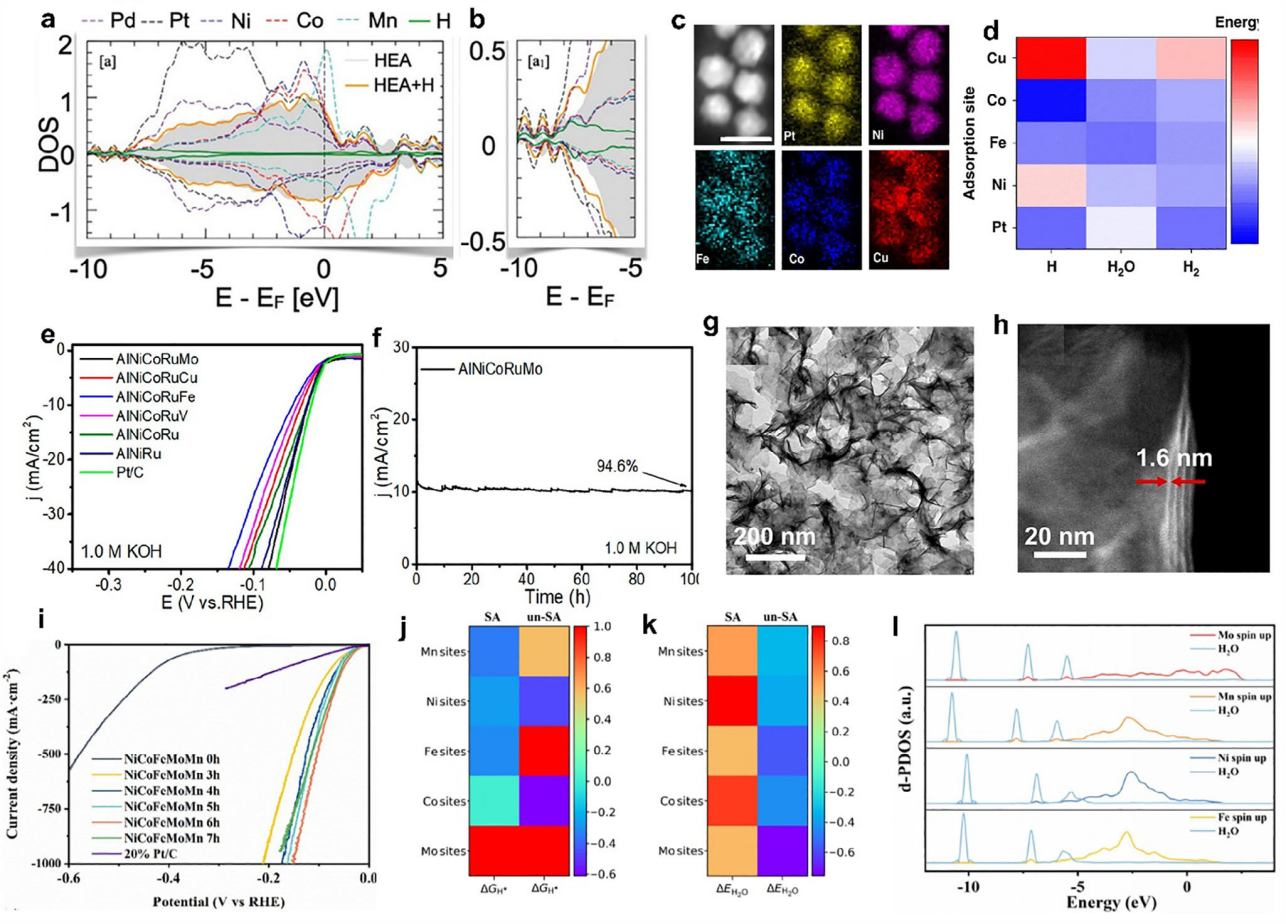
a) PDOS (states/eV‐atom) for HEA with and without hydrogen coverage. b) H‐1s states are weakly coupled with TM‐d states. a,b) Reproduced with permission.^[^ ^201^ ^]^ Copyright 2024, Wiley‐VCH. c,d) The elemental mapping and the binding energy mapping of Pt_18_Ni_26_Fe_15_Co_14_Cu_27_ nanoparticles (scale bar, 5 nm). c,d) Reproduced under the terms of the CC‐BY Creative Commons Attribution 4.0 International license.^[^ ^44^ ^]^ Copyright 2020, Springer Nature. e,f) HER polarization curves and long‐term durability test of AlNiCoRuMo. e,f) Reproduced under the terms of the CC‐BY Creative Commons Attribution 4.0 International license.^[^ ^204^ ^]^ Copyright 2020, American Chemical Society. g,h) TEM and HAADF‐STEM images of PdMoGaInNi nanosheets. g,h) Reproduced under the terms of the CC‐BY Creative Commons Attribution 4.0 International license.^[^ ^205^ ^]^ Copyright 2022, American Chemical Society. i) HER polarization curves for nanoporous NiCoFeMoMn. j–k) The colored Δ*G*_H*_ and Δ*E*_H2O_ comparisons of SA and un‐SA; the pure red area means this site is not easy to be adsorbed. l) The d‐PDOS plots of H_2_O and Mo, Mn, Ni and Fe adsorbed by H_2_O directly on un‐SA surface. i–l) Reproduced with permission.^[^ ^208^ ^]^ Copyright 2022, Elsevier.

Precious metal‐based materials, such as Pt, Ru, Pd, Rh, and Au, have long been considered the benchmark for HER, due to their outstanding intrinsic activity, favorable electronic structures, and robust stability. Alloying these noble metals together to form HEMs can lead to the development of unique structural and electronic properties. The synergistic interactions among multiple metal elements in HEMs may significantly enhance their catalytic performance for HER. PdPtRuRhAu HEAs with a particle size of 3.14 nm have demonstrated exceptional HER performance, with negligible activity loss over 100 h at high current densities of 500 and 1000 mA cm^−2^. The Pd‐Au bridge sites have been identified as the active sites for HER.^[^ ^44^ ^]^ Moreover, alloying these noble metals with transition metals is also an effective strategy to enhance HER performance while simultaneously reducing noble metal usage. The types and compositions of alloying transition elements are highly tunable, allowing for broad flexibility in catalyst design. A novel PtPdNiCoMn HEA has been prepared by room‐temperature electrodeposition method.^[^ ^201^ ^]^ Theoretical calculations revealed that the noble metals, surface Pt, and Pd sites exhibited optimized hydrogen adsorption capabilities, serving as the primary active sites. The Ni and Co species with their high density of states near the Fermi level facilitated the electron transfer and accelerate the HER kinetics (**Figure** **8**a,b). The PtPdNiCoMn HEA demonstrated an overpotential of only 22.6 mV at a current density of 10 mA cm^−2^ for alkaline HER. Except for noble metals as catalytic sites, the transition metal can also be the adsorption sites for HER. For instance, Pt combined with transition metals formed ultrasmall and uniformly sized Pt_18_Ni_26_Fe_15_Co_14_Cu_27_ HEAs (Figure 8c), in which Pt atoms acted as electron reservoirs, modulating the electronic structure of the surrounding transition metals to boost the alkaline HER.^[^ ^44^ ^]^ During the HER process, the water molecules initially adsorbed onto Fe sites, while the resulting *OH species are stabilized by adjacent hollow sites. H* adsorption preferentially occurred at hollow sites near Ni and Co (Figure 8d), and this synergistic interaction among the elements collaboratively enhanced the HER performance, achieving an ultra‐low overpotential (*η*_10_) of only 11 mV for alkaline HER.

Although low‐dimensional nanoparticles offer a high surface area and numerous active sites, they are susceptible to aggregation, which can compromise long‐term stability and catalytic efficiency. In contrast, HER electrocatalysts based on 1D, 2D, or 3D HEMs further expand their properties and kinetics. For instance, 1D HEM structures (e.g., nanowires or nanotubes), can not only facilitate rapid electron transport along their longitudinal axis, but also enhance reaction kinetics and structural stability.^[^ ^202^, ^203^ ^]^ The etched AlNiCoRuMo HEAs exhibited a 1D nanowire‐like morphology with diameters ranging from 20 to 100 nm, featuring ultrafine nanopores on the surface.^[^ ^204^ ^]^ The distinctive 1D nanowire structure provided efficient pathways for electron transport (Figure 8e), significantly enhancing the structural stability. Remarkably, the AlNiCoRuMo nanowires maintained 95.2% of their initial activity after 100 h of continuous HER testing, demonstrating the positive role of their structural stability in ensuring HER durability (Figure 8f). 2D HEMs with layered structures offer a balance between active site exposure and robust stability. The unique 2D nanosheets can also facilitate charge transfer and optimize ΔG_H*_, thereby accelerating HER kinetics. 2D PdMoGaInNi HEA nanosheets have been synthesized using a solution‐phase method, which exhibited a graphene‐like, wrinkled, single‐phase face‐centered cubic structure and an approximate thickness of 1.6 nm (Figure 8g,h).^[^ ^205^ ^]^ These nanosheets demonstrated an exceptionally low overpotential of merely 13 mV at a current density of 10 mA cm^−2^, along with excellent long‐term stability for over 200 h at 100 mA cm^−2^ in a proton exchange membrane water electrolyzer. This significant durability and performance improvement can be attributed to the introduction of Pd to protect this unique 2D layered structure, which is conducive to rapid mass transport during the reaction. Moreover, 3D porous frameworks further enhance mass transport and active site accessibility.^[^ ^161^ ^]^ Their interconnected porous networks improve gas diffusion and electrolyte penetration, enabling efficient catalysis at industrial‐scale current densities with low overpotentials. For instance, Pt (Co/Ni) MoPdRh HEA nanoflowers, assembled from ultrathin nanosheets (≈1.68 nm), exhibited remarkable advantages in alkaline HER.^[^ ^205^ ^]^ Furthermore, the mesoporous 3D structures of PtPdRuMoNi nanospheres achieved by low‐temperature triblock copolymer‐assisted wet‐chemical method featured a mesopore size of 10–12 nm and an inter‐pore distance of ≈16 nm.^[^ ^175^ ^]^ The mesoporous structure highly exposed active sites and achieves a mass activity of 167 A g^−1^ at an overpotential of 30 mV, far exceeding that of commercial Pt/C (34 A g^−1^). The high entropy properties enable electron transfer among the multi‐elements, optimizing the d‐band center of Pt, Pd, and Ru, and effectively regulating the adsorption energy of HER intermediates.

#### 4.1.2 Pristine Non‐Noble Metal‐Based HEMs

While precious metal‐based HEMs offer excellent HER catalytic performance, their high cost and scarcity have driven the search for alternative materials. Non‐precious metal catalysts have emerged as promising candidates due to their natural abundance, cost‐effectiveness, and tunable electronic structures. Non‐precious metal‐based HEMs for HER primarily include transition metals such as Ni, Co, Fe, Mo, W, V, Mn, and Cr. Different elements are generally suited to different types of HEMs and contribute distinct properties that influence HER performance.^[^ ^206^, ^207^ ^]^ For instance, Ni, Co, and Fe‐based alloys have been widely used as alkaline HER electrocatalysts due to their excellent electrical conductivity and moderate hydrogen binding energy. Mo and W‐based sulfides or carbides can form Pt‐like active sites and exhibit excellent HER activity. V, Ti, Cr, etc. contribute to electronic modulation and structural stability in HEMs. Integrating transition metals with highly matched atomic radii into HEMs can generate homogeneous solid solutions, leveraging cocktail effects to boost catalytic performance. For instance, the NiCoFeMoMn HEA nanoparticles achieved a remarkably low overpotential of ≈14 mV at a current density of 10 mA cm^−2^, significantly outperforming the commercial Pt/C electrode (32 mV) (Figure 8i).^[^ ^208^ ^]^ Among the multiple elements, Ni, Co, and Fe were the main active centers for HER, promoting hydrogen adsorption (Δ*G*_H* _= −0.05, −0.03, and 0.01 eV) and the adsorption energy of H_2_O (Δ*G*_H2O _= −0.15, −0.19, and −0.3 eV). (Figure 8j–l) Mo contributed by enhancing water adsorption and dissociation through strong orbital interactions between its d‐orbital spin density and the O 2p orbital of H_2_O, thereby further accelerating HER kinetics. In addition to the active elements (Fe, Co, and Ni) serving as HER active sites, the synergistic effects within HEMs can transform traditionally inactive elements (e.g., Cu) into highly competitive active sites for HER. A highly active FeCoNiCuMn HEA nanoparticle was developed through a polymer fiber nanoreactor strategy.^[^ ^110^ ^]^ The strong local electronic interactions among the multiple metal sites within the FeCoNiCuMn HEA enabled Cu to serve as the primary active site for alkaline HER. This is attributed to its lowest H_2_O dissociation barrier (0.54 eV) and the closest H* adsorption‐free energy (−0.085 eV) to the ideal value of 0 eV. The FeCoNiCuMn HEA demonstrated an overpotential of 281 mV at a high current density of 100 mA cm^−2^, surpassing the performance of commercial Pt/C catalysts (302 mV).

**Figure 9:**
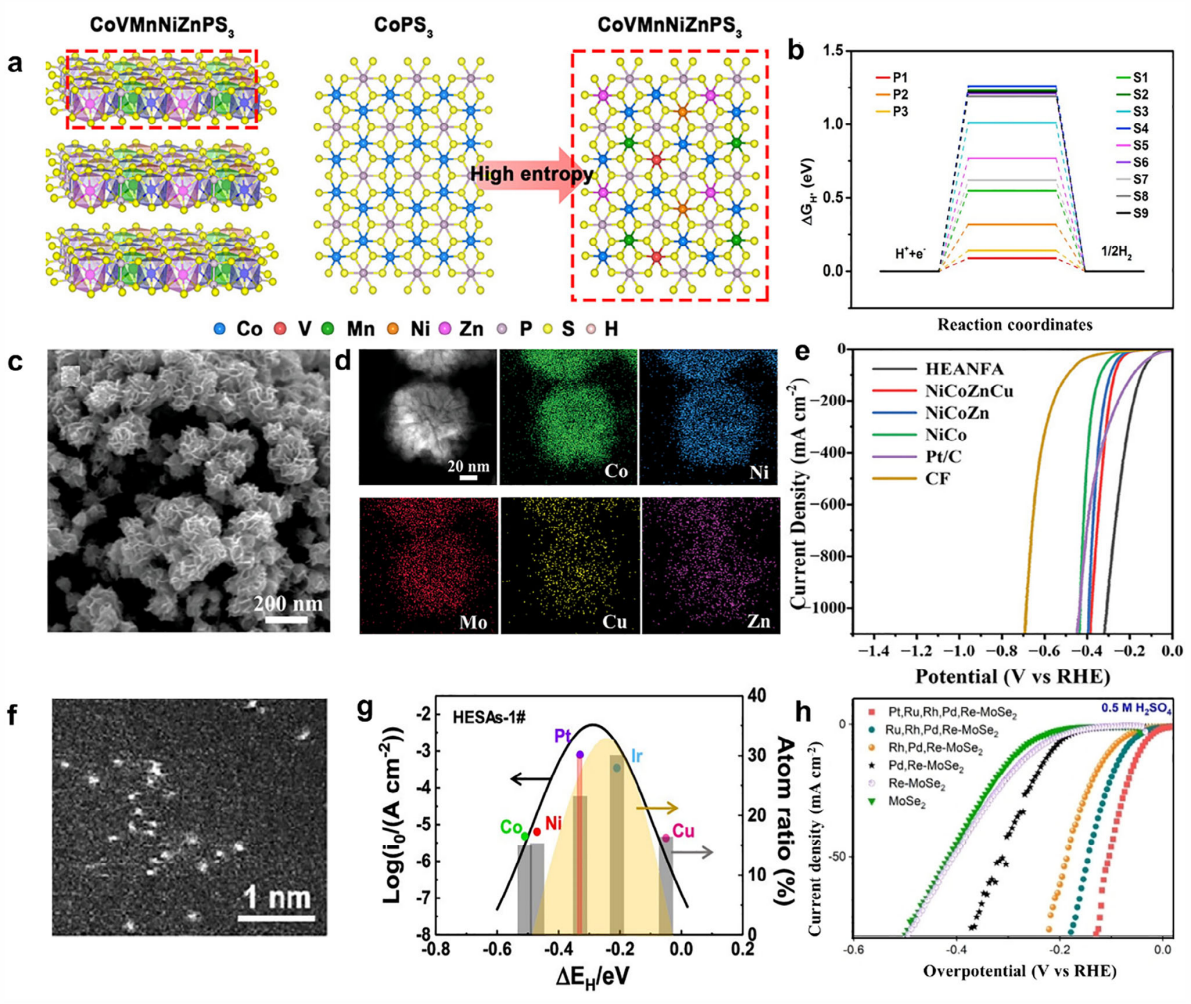
a) The CoVMnNiZnPS_3_ crystallizes in a monoclinic structure (space group C2/m), with top‐view illustrations demonstrating the structural evolution from pristine CoPS_3_ to CoVMnNiZnPS_3_. b) HER free‐energy diagram of different sites in CoVMnNiZnPS_3_. a,b) Reproduced with permission.^[^ ^88^ ^]^ Copyright 2022, American Chemical Society. c) SEM image and d) HAADF‐STEM image and EDS mapping of NiCoMoZnCu HEA nanoflower. e) LSV curves of HEANFA and other comparative catalysts in 1.0 M KOH solution. c–e) Reproduced with permission.^[^ ^212^ ^]^ Copyright 2024, Wiley‐VCH. f) HAADF STEM images of Pt SAs in CB. g) Calculated distribution plots for PtIrCuNiCo HESACs. f,g) Reproduced with permission.^[^ ^213^ ^]^ Copyright 2023, American Chemical Society. h) Polarization curves of PtRuRhPdRe‐MoSe_2_ and other comparative catalysts in acidic and electrolytes. h) Reproduced with permission.^[^ ^106^ ^]^ Copyright 2024, Wiley‐VCH.

Similar to noble metal‐based HEMs, transitioning from nanoparticles to higher‐dimensional structures (1D, 2D, and 3D) further accelerates the reaction kinetics of HEMs for HER.^[^ ^33^, ^209^, ^210^, ^211^ ^]^ 1D nanowire high‐entropy CoZnCdCuMnS exhibited a needle‐like morphology.^[^ ^114^ ^]^ This unique structure not only shortens the diffusion pathways of reactants and products but also increases the exposure of active sites. Specifically, Co served as the primary active sites, and the other elements efficiently modulate the electronic distribution of the active sites, thereby optimizing the H* adsorption energy and achieving high HER performance. In 1 m KOH solution, CoZnCdCuMnS demonstrated excellent electrocatalytic performance for HER, achieving a current density of 10 mA cm^−2^ at an overpotential of 173 mV. 2D materials offer several unique advantages due to their ultrathin, atomically layered structures. For instance, Co_0.6_(VMnNiZn)_0.4_PS_3_ high entropy 2D nanosheets with a thickness of ≈2.8 nm were synthesized by combining conventional solid‐phase reaction and ultrasonic‐assisted technology (**Figure** **9**a).^[^ ^88^ ^]^ The nanosheet structure enabled it to expose abundant active sites, achieving a low overpotential of 65.9 mV and a Tafel slope of 65.5 mV dec^−1^ for alkaline HER. Moreover, mechanistic investigations suggest that Mn sites lowered the energy barrier for water dissociation, thereby facilitating the Volmer step, while the edge S sites and substrate P sites served as active sites for hydrogen adsorption, collaboratively enhancing the alkaline HER performance (Figure 9b). The 3D hierarchical architectures with interconnected conductive networks can efficiently expose active sites while maintaining mechanical integrity. Benefiting from the advantage of a 3D unique structure, NiCoMoZnCu HEA nanoflower array (HEANFA) exhibited outstanding HER activity, requiring only 242.9 and 307.5 mV to achieve industrial‐level current densities of 500 and 1000 mA cm^−2^, considerably outperforming the commercial Pt/C catalyst (365 and 437 mV) (Figure 9c–e).^[^ ^212^ ^]^ DFT calculations were performed to determine the water dissociation energy barriers by investigating each element in NiCoMoZnCu HEANFA as the active site. The results indicate that the H_2_O dissociation energy barriers at Co, Mo, Cu, Ni, and Zn sites are 0.51, 0.43, 0.76, 0.61, and 0.39 eV, respectively, all significantly lower than that of pure Pt (0.95 eV). This suggests that the HEANFA surface facilitated the H_2_O dissociation into H* during the Volmer step. Additionally, the free energy of the Heyrovsky step at various HEANFA surface metal sites (−0.12, 0.11, −0.05, and −0.23 eV) is also markedly lower than that on Pt (0.23 eV), optimizing Δ*G*_H*_ closer to zero and thereby enhancing HER activity.

#### 4.1.3 High Entropy Single Atoms Catalysts

High entropy single‐atom catalysts (HESACs) maximize the utilization of metal atoms and optimize the geometric structure of active metals for HER. The metal cations adjacent to the active metal act as promoters to regulate the electronic structure, orbital configuration, and catalytic activity of the active metal through non‐bonding and interaction. Due to the high energy of isolated metal species, the HESACs require substrates to anchor and stabilize these multiple isolated metal atoms, including carbon (e.g., carbon black, carbon quantum dots, and graphene.) and non‐carbon substrates (transition metal sulfides, oxides, etc.). For instance, a one‐step laser implantation strategy has been used to anchor Pt, Ir, Cu, Ni, and Co metal atoms on defective carbon black (Figure 9f).^[^ ^213^ ^]^ The defects on carbon black acted as anchoring sites to load 42 wt.% of multiple atoms of high quality. The cocktail effect results in a mass activity of PtIrCuNiCo HESAC with low noble metal loading 11 times higher than that of commercial Pt/C. Combining theoretical calculations and experimental results, it is found that the distribution of each atom ratio in PtIrCuNiCo HESACs is closer to the activity distribution of the corresponding metal in the HER volcano diagram, thus achieving excellent catalytic activity (Figure 9g). Furthermore, the chalcogens (S, Se) in 2D transition metal dichalcogenides (TMDs) have lone pairs of electrons and certain electronegativity, which can interact with metal atoms to achieve a stable effect. In addition, the abundant vacancies on the surface of TMDs can act as anchoring sites to stabilize the metal atoms. Through the substrate‐mediated strategy to control the reversible redox reaction at the interface of TMDs and transition metal ions, high‐atom‐density PtRuRhPdRe‐MoSe_2_ (HESAs‐01) was obtained.^[^ ^106^ ^]^ Chemically synthesized MoSe_2_ contained electron‐donating Se vacancies that endowed redox capability, whereas the Mo vacancies were generated upon the introduction of Pt, Ru, Rh, Pd, or Re ions. The incorporated metal cations are reduced by MoSe_2_ and spontaneously migrate to occupy the Mo vacancies, coordinating with Se without metallic bond formation (Figure 9h). In both acidic and alkaline media, HESAs‐01 showed excellent HER activity (*η*_10,acid_ = 32 mV, *η*_10,alkaline_ = 35 mV), which is better than the state‐of‐the‐art single‐atom materials. The multi‐metal single atoms enhanced the metal–support interaction and further adjust the electronic state of MoSe_2_, stimulating high HER activity and stability.

#### 4.1.4 High Entropy Intermetallic

Compared to the traditional disordered HEMs, HEI possess higher structural stability, more precise electronic regulation, more effective site isolation effect, and multifunctionality. Therefore, HEI inherits the advantages of HEMs and intermetallic compounds to be promising candidates for HER. When the disordered HEMs transform into an ordered HEI structure, it is usually accompanied by a change in the electronic structure, for example, adjusting the d‐band center. For example, changing the annealing temperature and time can effectively adjust the order degree of Pt_4_FeCoCuNi nanocrystals to achieve highly ordered, partially ordered, and fully disordered samples.^[^ ^51^ ^]^ Compared with partially ordered and fully disordered Pt_4_FeCoCuNi, in the highly ordered sample, the d‐band center was positioned closest to the Fermi level, which significantly improved the binding affinity of HER intermediates and boosted catalytic efficiency. Moreover, the alternating stacking of Pt in the highly ordered Pt_4_FeCoCuNi crystal structure promoted H* coupling and Fe/Co/Cu/Ni species favor water dissociation, synergistically enhancing the HER activity. As a result, the highly ordered Pt_4_FeCoCuNi only took 20 mV to reach *j* of 10 mA cm^−2^, which is much lower than the other two counterparts (32 and 47 mV, respectively). In addition to the annealing condition affecting the order degree of HEMs, the metal species also promote the transformation from disordered to ordered HEI structure. For example, the introduction of Fe induced the change of PtCuPdAg from the L1_1_ phase (PCPAF‐HEA/C) to the L1_0_ intermetallic phase (PCPAF‐HEI/C).^[^ ^214^ ^]^ In 0.5 m H_2_SO_4_ solution, PCPAF‐HEI/C showed high specific activity (SA = 34.9 mA cm^−2^) and low Tafel slope (29 mV dec^−1^), which is significantly better than that of PCPAF‐HEA/C (SA = 26.16 mA cm^−2^, Tafel slope = 38 mV dec^−1^) and Pt/C (SA = 1.681 mA cm^−2^, Tafel slope = 35 mV dec^−1^). The excellent alkaline HER activity of PCPAF‐HEI/C can be attributed to the stronger d–d interaction and high entropy stabilization effect in its ordered structure compared with PCPAF‐HEA/C. Compared with pure Pt (−0.587 eV), the H* of the Pt site in PCPAF‐HEI/C was optimized with a Δ*G*_H*_ of −0.434 eV, which further alleviates the excessive binding of hydrogen on the surface and accelerates the desorption of H_2_.

#### 4.1.5 Defective HEMs

**Figure 10:**
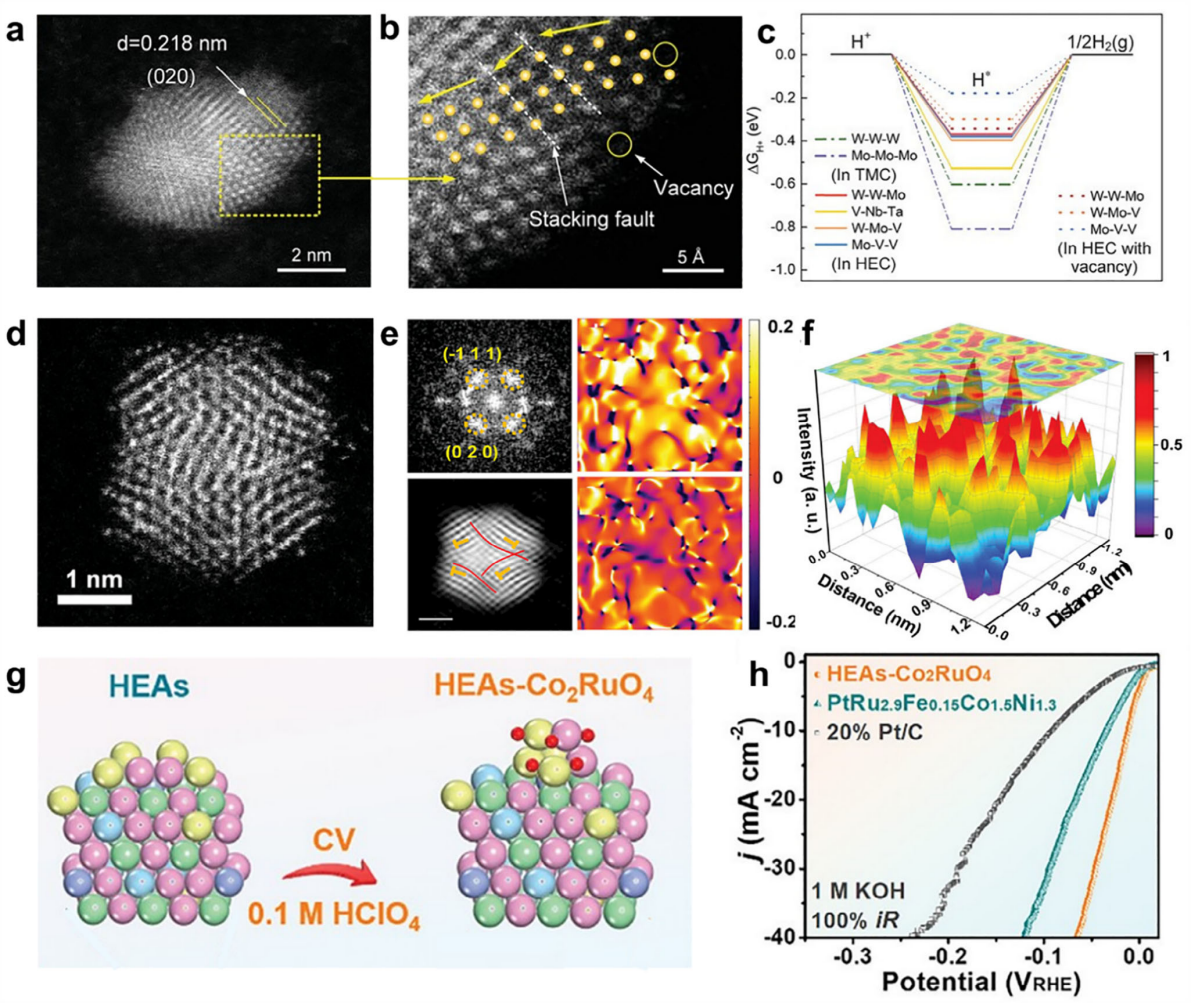
a,b) HAADF‐STEM images and the zoom‐in sites of HECNPs. c) Gibbs free‐energy diagram of HER. a–c) Reproduced with permission.^[^ ^100^ ^]^ Copyright 2022, Wiley‐VCH. d) HAADF‐STEM image of an individual IrRuRhMoW. e) fast Fourier transform (FFT) pattern of the region in (d). f) 3D topographic image of atomic sites and relative intensity distribution of IrRuRhMoW HEA NPs. d–f) Reproduced with permission.^[^ ^217^ ^]^ Copyright 2024, Wiley‐VCH. g) The structure reconstruction of HEAs‐Co_2_RuO_4_. h) LSVs of 5% HEAs‐Co_2_RuO_4_ and the comparison materials. g,h) Reproduced with permission.^[^ ^181^ ^]^ Copyright 2025, Wiley‐VCH.

Defects play a crucial role in modifying electronic structures, increasing active sites, and improving reaction kinetics. Common types of defects include vacancies, dislocations, stacking faults, and voids, all of which play a significant role in boosting the HER catalytic performance.^[^ ^215^ ^]^ Wire electrical discharge machining (WEDM) technique produces ultra‐high temperatures followed by rapid quenching, enabling the transformation of bulk (MoWVNbTa)C into sub‐10 nm nanoparticles.^[^ ^100^ ^]^ Interestingly, high‐entropy carbide nanoparticles exhibit a high density of defects, including steps, vacancies, and stacking faults, resulting from the ultrafast quenching process (**Figure** **10**a,b). DFT calculations were conducted using vacancy defects as a representative model. Sites containing Mo or W species show low ΔG_H*_, suggesting they serve as active sites for acidic HER. The DFT calculation took the vacancy defect as a representative. Sites containing Mo or W species show low Δ*G*_H*_, suggesting they served as active sites for acidic HER (Figure 10c). Furthermore, when surrounded by vacancy defects, these sites exhibited more favorable Δ*G*_H*_ values compared to those in the intact crystal structure, due to a moderated d‐band center value and the shielding effect of the vacancy for electron transfer. The unique defect‐rich microstructure and high configurational entropy synergistically boosted the acidic HER activity, exhibiting a low overpotential of 156 mV at 10 mA cm^−2^, outperforming monometallic carbides (238–495 mV) and bulk HECs (402 mV). These findings further reinforce that defect‐rich structures, particularly those incorporating vacancies and lattice distortions, play a crucial role in enhancing HER activity.

Defects engineering high‐entropy metallic glass (HEMG) surfaces can also dramatically enhance HER efficiency. Significant lattice distortions and stacking faults have been introduced to the nanoporous (FeCoNiB_0.75_)_97_Pt_3_ HEMG with a nanocrystalline surface structure, which optimized atomic configurations and modulated electronic interactions.^[^ ^216^ ^]^ Moreover, such lattice distortion significantly enhances the adsorption energy of H_2_O at Fe and Co sites in the stacking faults structure. During the water adsorption step, the H_2_O adsorption energies at Fe and Co sites in the stacking faults structure reached −0.70 and −0.44 eV, respectively, substantially higher than those on the Pt (111) surface. This indicates that defects facilitate the optimization of atomic configurations and promote water adsorption. In the HER process, sites such as Pt top, Pt‐Co bridge, and Pt‐Ni‐Ni hollow exhibit favorable Δ*G*_H*_values, serving as critical active sites. Notably, the Pt sites coordinated with B atoms demonstrate Δ*G*_H*_ values close to zero (0.0074 and 0.0312 eV), contributing significantly to the enhancement of HER performance. The resulting defect‐rich architecture enabled ultralow overpotentials of 104 mV at 1000 mA cm^−2^ for HER under alkaline conditions, with exceptional long‐term stability exceeding 200 h at 100 mA cm^−2^. The lattice distortions can also result in unsaturated oxygen sites to enhance the surface oxophilicity. Due to the high entropy effect, the introduction of Mo and W caused local tensile and compressive microstrain in IrRuRhMoW HEA sub‐nanoparticles (Figure 10d–f).^[^ ^217^ ^]^ These strain defects generated a large number of unmatched O sites on the surface of IrRuRhMoW HEA, resulting in O defects that effectively reduce the d‐band center. In 0.1 m KOH, IrRuRhMoW HEA achieved a turnover frequency (TOF) of 5.93 H_2_ s^−1^ at an overpotential of 70 mV, which is 4.6 times that of commercial Pt/C catalysts. Therefore, precise control of defects is crucial to improve HER catalytic activity.

#### 4.1.6 Surface Reconstructed HEMs

Surface reconstruction in HEMs has emerged as a key strategy for enhancing HER performance and the reconstructed layers can optimize hydrogen adsorption energy, promote efficient water dissociation, and introduce additional active sites. Reconstruction can be induced by electrochemical processes or external energy input. For instance, self‐reconstructed PtRu_2.9_Fe_0.15_Co_1.5_Ni_1.3_‐Co_2_RuO_4_ HEA has been achieved via electrochemically induced oxidation.^[^ ^181^ ^]^ During cyclic voltammetry activation in acidic media, the formation of an interfacial Co_2_RuO_4_ phase on the HEA surface significantly reduced the Helmholtz layer, promoting enhanced electrolyte interaction (Figure 10g). This structural transition facilitated H_2_O adsorption at Co^2+^/Ru^3+^ sites while weakening H* adsorption, thereby accelerating alkaline HER kinetics. This hybrid structure demonstrated an exceptionally low overpotential of 11.8 mV at 10 mA cm^−2^, with a current density of 41.8 mA cm^−2^ at 0.07 V versus RHE, outperforming conventional Pt‐based catalysts (Figure 10h). External energy input, such as laser etching, could also effectively trigger self‐reconstruction and optimize the catalytic activity of HEMs. The NiFeCrVTi HEA underwent laser processing to generate a hierarchical porous structure, which not only facilitated electron transfer but also exposed abundant active sites.^[^ ^218^ ^]^ Laser etching also locally induced oxidation, forming surface NiOOH/NiO_x_ layers, reducing the adsorption and dissociation energy barriers of H_2_O. Similarly in seawater, the optimized Nickel‐based high‐entropy alloy etched by 30 W laser (Ni‐HEA‐30) catalyst exhibited remarkable HER performance, achieving overpotentials of 37.9 mV in NaCl solution and 55.9 mV in natural seawater at a current density of 10 mA cm^−2^, surpassing conventional non‐precious electrocatalysts. The strong interaction between the high‐entropy matrix and the in situ reconstructed oxide layer played a crucial role in stabilizing active sites, further improving catalytic durability. These findings highlight the potential of surface reconstruction strategies in HEMs, offering new insights into rational catalyst design for high‐performance electrocatalysis.

#### 4.1.7 Functionalized HEMs

**Figure 11:**
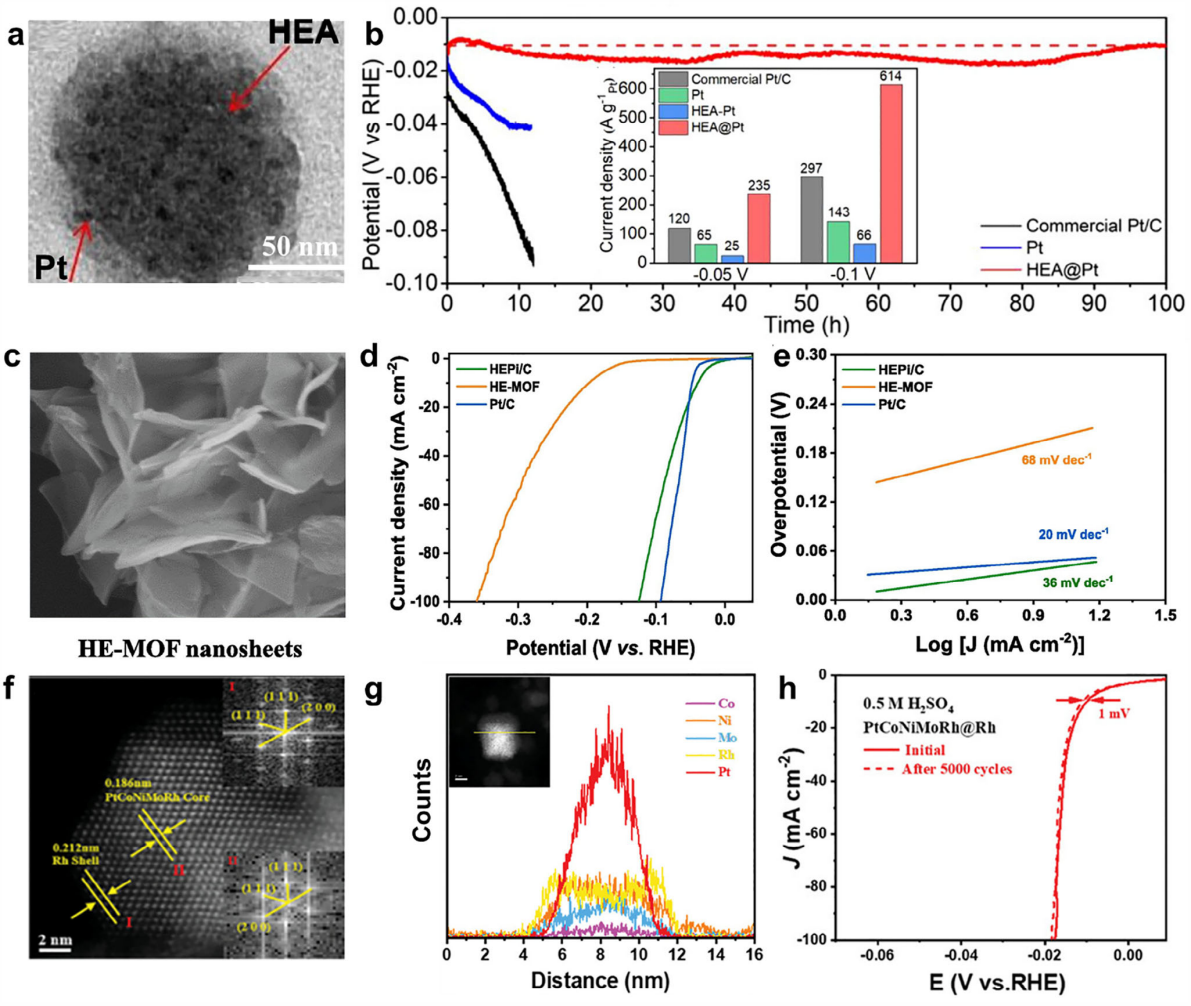
a) The TEM images of HEA@Pt. b) The stability tests of Pt, commercial Pt/C and HEA@Pt, the inset is the mass activity of the comparative catalyst and HEA@Pt. a,b) Reproduced under the terms of the CC‐BY Creative Commons Attribution 4.0 International license.^[^ ^225^ ^]^ Copyright 2022, Wiley‐VCH. c) SEM image of HEPi/C hybrid nanosheets. d,e) HER polarization curves and overpotential of HE‐MOF nanosheets and comparative catalysts. c–e) Reproduced with permission.^[^ ^119^ ^]^ Copyright 2022, Elsevier. f) Linear scan spectrum of PtCoNiMoRh@Rh. g) HR‐STEM image, the inset is the fast Fourier transform mode of the shell–core region. h) LSV polarization curves of PtCoNiMoRh@Rh before and after 5000 cycles in acidic medium. f–h) Reproduced with permission.^[^ ^232^ ^]^ Copyright 2024, Wiley‐VCH.

While pristine, defective, surface reconstructed HEMs provide a versatile platform for water splitting, further functionalization can maximize their catalytic capability.^[^ ^219^, ^220^, ^221^, ^222^, ^223^ ^]^ Strategies such as incorporating single atoms, clusters, nanoparticles, surface engineering, and forming hybrids, produce cocktail effects and have significantly optimized their performance as HER electrocatalysts. For instance, Mo single atoms modified PdPtNiCuZn HEA nanosheets promoted strong electron transfer to adjacent Pt atoms, increasing the electron density of Pt atoms while rendering Mo electron‐deficient.^[^ ^224^ ^]^ This modification also shifted the d‐band center of the strained Mo_1_‐PdPtNiCuZn system upward, reducing antibonding orbital occupancy and significantly altering the binding energies of key reaction intermediates. Atomic clusters or nanoparticles incorporation to HEMs can also boost the HER catalytic performance. A HEA@Pt hybrid catalyst with Pt clusters uniformly stabilized on a FeCoNiCu HEA core has been prepared by ultra‐fast shock synthesis and galvanic replacement.^[^ ^225^ ^]^ The unique core–shell structure and synergistic effect between atomic cluster and HEA led to a remarkable HER mass activity of 235 A g_Pt_^−1^ at −0.05 V, which is 9.4 times higher than homogeneous HEA‐Pt and 1.9 times higher than commercial Pt/C (**Figure** **11**a,b). Additionally, the HEA@Pt catalyst exhibited outstanding long‐term stability, with negligible performance decay over 100 h of operation, further demonstrating the effectiveness of HEA support in stabilizing Pt active sites. The integration of HEMs with non‐metallic materials, such as carbon‐based structures and metal–organic frameworks (MOFs), has also garnered significant attention.^[^ ^226^, ^227^ ^]^ High‐entropy phosphate/carbon (HEPi/C) hybrid nanosheets achieved by high‐temperature phosphidation with high‐entropy MOF nanosheets as precursors.^[^ ^119^ ^]^ The HEPi/C hybrid structure integrated the unique electronic states and surface properties of high‐entropy phosphates with the excellent conductivity and structural stability of carbon, demonstrating remarkable acidic HER activity (an overpotential of only 40 mV at 10 mA cm^−2^ and Tafel slope of 38 mV dec^−1^) (Figure 11c–e). DFT calculations indicate that the d‐band center of high‐entropy phosphates shifted significantly downward compared to single‐metal catalysts, which helps weaken the adsorption energy of H* and thus enhancing the HER activity.

Integrating HEMs with functional components forming interfaces, including metal layers, oxides, carbides, and phosphides, has emerged as a promising strategy to enhance HER performance.^[^ ^228^, ^229^, ^230^ ^]^ By leveraging the synergistic effects between different phases, hybrid catalysts can effectively modulate active sites, leading to superior catalytic activity. A dual‐phase PtNiCuMnMo HEA with both FCC and BCC phases has been prepared via arc melting.^[^ ^231^ ^]^ X‐Ray Diffraction and Transmission electron microscope (TEM) analyses revealed that the unique atomic coordination environment at phase boundaries induced lattice mismatch, generating strain fields that modified the electronic structure and charge transfer behavior. The coexistence of FCC and BCC phases not only enhanced the electrical conductivity of the material but also improved hydrogen adsorption energy, further facilitating alkaline HER performance with an overpotential of 44 mV for a current density of 10 mA cm^−2^. Moreover, the core–shell architectures PtCoNiMoRh@Rh have been achieved via a one‐step wet chemical method, which effectively protected the inner transition metals, preventing dissolution and maintaining structural integrity under harsh electrochemical conditions (Figure 11f,g).^[^ ^232^ ^]^ The catalysts demonstrate remarkable stability with the overpotential increased by only 1 mV after 5000 cycles (Figure 11h). Moreover, the mass activities of PtCoNiMoRh@Rh were 5.8, 2.79, and 91.8 times higher than that of Rh/C in acidic, neutral, and alkaline electrolytes, indicating the modification effect of HEA core with the Rh shell in enhancing HER catalytic performance. Incorporating active components into HEMs creates heterostructures inducing cocktail effects and more exposed active sites. The stabilization of FeCoNiCrMn on MoS_2_, forming 1D MoS_2_@HEP hybrid nanowires, induced strong electron transfer from MoS_2_ to the high‐entropy phase.^[^ ^233^ ^]^ This electronic modulation effectively lowers the free energy of hydrogen adsorption (Δ*G*_H*_) at the edge active sites of MoS_2_, thereby significantly enhancing the HER performance. The MoS_2_@HEP hybrid nanowires exhibited outstanding alkaline HER performance, achieving an overpotential of 71 mV at *j* of 10 mA cm^−2^.

### 4.2 Oxygen Evolution Reaction

The OER is a kinetically sluggish and complex process involving four proton‐coupled electron transfers, which is a critical bottleneck in electrochemical water splitting. Depending on the acidity and alkalinity of the electrolyte solution, the OER undergoes different reaction processes. In alkaline conditions, the hydroxyl group as the reactant is gradually oxidized to form O_2_ and H_2_O. In contrast, in acidic electrolytes, water molecules are oxidized to generate O_2_ and H^+^, a process that is more complex and requires breaking the strong O─H covalent bonds in H_2_O, leading to slower reaction kinetics. There are three primary catalytic mechanisms for OER, including the adsorbate evolution mechanism (AEM), the lattice oxygen‐mediated mechanism (LOM), and the oxidation pathway mechanism (OPM). In AEM, oxygen evolution occurs through the stepwise adsorption and transformation of intermediates such as *O, *OH, and *OOH. In contrast, the LOM involves the participation of lattice oxygen to enable direct O─O bond formation, while the OPM facilitates the coupling of adjacent *O intermediates without the formation of *OOH. Each of these pathways has unique advantages and challenges. The gap between the adsorption energies of *OH and *OOH in AEM (3.2 eV) limits the OER overpotential and shows poor catalytic activity. The participation of lattice oxygen in LOM can significantly reduce the overpotential, but the generated O vacancies aggravate the collapse of the catalyst structure and may show poor stability. OPM has high requirements for the atomic structure of the designed catalyst and can only be triggered at the optimal atomic spacing. The above issues further highlight the importance of specially tailored catalysts to enhance the OER catalytic activity.

#### 4.2.1 Pristine Noble Metal‐Based HEMs

**Figure 12:**
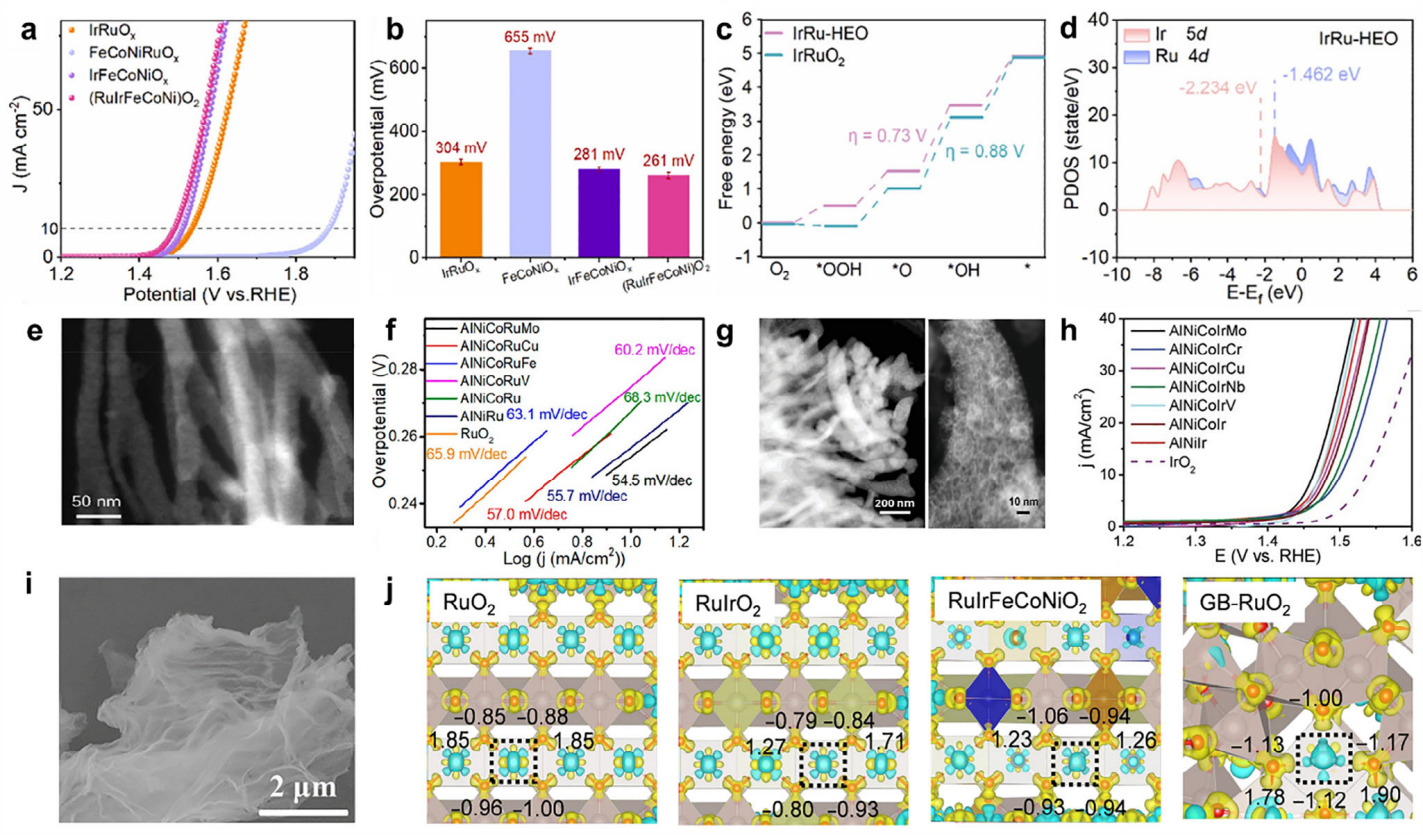
a) LSV polarization curves of IrRu‐HEO and comparative samples in acidic medium. b) Overpotential comparison. c,d) OER Gibbs free energy diagrams and PDOS for IrRu‐HEO. a–d) Reproduced with permission.^[^ ^239^ ^]^ Copyright 2024, Elsevier. e) HRTEM image of the dealloyed AlNiCoRuMo nanowires. f) Tafel curves of prepared samples. e,f) Reproduced with permission.^[^ ^204^ ^]^ Copyright 2020, American Chemical Society. g) STEM images of AlNiCoIrMo HEA. h) OER polarization curves of all these prepared samples. g,h) Reproduced with permission.^[^ ^160^ ^]^ Copyright 2025, Elsevier. i) SEM images of (FeCoNiCrMn)_3_O_4_. j) Top view of the charge density difference of these four models. i,j) Reproduced with permission.^[^ ^241^ ^]^ Copyright 2024, Royal Society of Chemistry.

Noble‐metal‐based HEMs, incorporating elements like Ru, Ir, Rh, and Pt, modulate electronic structures and oxygen adsorption energies to enhance catalytic efficiency and stability. The diverse structural configurations of these materials, spanning from atomic‐scale clusters to extended frameworks, enable precise control over active site density and reaction kinetics. By systematically tuning the composition and morphology of HEMs, it can pave the way for the rational design of next‐generation electrocatalysts. 0D high‐entropy nanoparticles have garnered significant attention for OER due to their ultra‐small size, which maximizes the number of exposed active sites and facilitates electron transfer.^[^ ^234^, ^235^, ^236^, ^237^, ^238^ ^]^ Ru‐containing HEMs effectively improve the instability of traditional Ru‐based materials in acidic OER. For example, single‐phase solid solution RuMnFeMoCo nanoparticles (diameter of ≈10–20 nm) were prepared using carbon felt (CF) as the substrate under a rapid Joule heating synthesis method.^[^ ^20^ ^]^ In 0.5 m H_2_SO_4_ electrolyte, RuMnFeMoCo exhibited an overpotential of 170 mV at 10 mA cm^−2^ and maintained stable operation for 1000 h under these conditions. The excellent OER activity and stability of RuMnFeMoCo were due to the cocktail effect of multiple metals, which weakens the adsorption energy of oxygen‐containing intermediates and promotes the formation of *OOH.^[^ ^132^ ^]^ The post‐characterization further confirms that RuMnFeMoCo maintained the geometric and electronic structure, showing excellent durability. The corrosion resistance of Ir provides an option for designing HEM catalysts for acidic OER. IrFeCoNiCu‐HEA nanoparticles (average size ≈20–200 nm) were synthesized on carbon paper using microwave‐assisted impact synthesis. During the electrochemical activation, the surface transition metals of IrFeCoNiCu‐HEA gradually dissolved in acidic conditions, forming an Ir‐rich layer with a thickness of ≈2–6 nm on the surface of the nanoparticles. The internal undissolved transition metals have an electronic regulation effect on the surface active Ir, achieving a low overpotential (*η*) of 302 mV for a current density of 10 mA cm^−2^ in 0.1 m HClO_4_. The coexistence of Ir and Ru in HEOs can further promote the acidic OER. The (RuIrFeCoNi)O_2_ (IrRu‐HEO) nanoparticles (50–60 nm) synthesized by molten salt oxidation method achieved an overpotential of 261 mV for a current density of at 10 mA cm^−2^ in 0.1 m HClO_4_, which was better than other metal oxides IrRuO_x_ (304 mV), FeCoNiO_x_ (655 mV) and IrFeCoNiO_x_ (281 mV) (**Figure** **12**a,b).^[^ ^239^ ^]^ The high OER activity of IrRu‐HEO is attributed to the complex metal─O bonds, which shift the d‐band center of Ir and Ru downward under the regulation of multiple components, reducing the OER reaction energy barrier (Figure 12c,d).

Similar to the HER, OER electrocatalysts based on 1D, 2D, or 3D HEMs further expand their properties and kinetics. For instance, the inherent structural flexibility of nanowires provides enhanced durability during prolonged OER cycling. High entropy nanowires (HE‐NWs) composed of noble metals can induce complex electronic interactions between different components in a single phase with multiple metals. For example, five‐element AlNiCoRuMo NWs were prepared by melt spinning combined with alkali treatment, where the diameter of the nanowires was ≈20–100 nm.^[^ ^204^ ^]^ AlNiCoRuMo NWs have stronger electronic interactions, making their Tafel slope in alkaline electrolytes as low as 54.5 mV dec^−1^, revealing fast OER kinetics (Figure 12e,f). The introduction of metal Mo to NWs makes the *e*_g_ orbital occupancy of the active site Ru closer to 1, which optimizes the adsorption strength of multiple intermediates in the OER process. Similar to the performance of HE‐NWs containing noble metal Ru, noble metal HE‐NWs containing Ir also showed outstanding OER activity. The AlNiCoIrMo HEA has a porous nanoligament structure (diameter of ≈100 nm), which promotes mass and gas transport, and active site exposure resulting in a high electrochemically active surface area of 57.6 m^2^ g^−1^ for OER (Figure 12g).^[^ ^160^ ^]^ In acidic media, the overpotentials of AlNiCoIrMo HEA at a current density of 10 or 20 mA cm^−2^ are only 233 and 255 mV, respectively, which is significantly lower than that of IrO_2_ (*η*_10_ = 300 mV, *η*_20_ = 330 mV) (Figure 12h). The enhanced OER activity of the AlNiCoIrMo HEA is primarily attributed to the covalent nature of the Ir─O bond, which is significantly promoted by Mo doping.

The high surface‐to‐volume ratio of 2D high‐entropy nanosheets ensures efficient mass transport, while their unique electronic states‐tuned by multicomponent interactions‐enhance the adsorption and desorption kinetics of key reaction intermediates.^[^ ^26^, ^240^ ^]^ 2D (FeCoNiMoRu)_3_O_4_ HEOs prepared by rapid Joule heating and self‐sacrificial template method, possessed a large active surface area, which is conducive to shortened ion transport paths (Figure 12i).^[^ ^241^ ^]^ (FeCoNiMoRu)_3_O_4_ showed excellent catalytic activity (*η*_10_ = 199 mV, *η*_100_ = 266 mV) for OER, which was better than (FeCoNiCrMn)_3_O_4_ (*η*_10_ = 263 mV, *η*_100_ = 364 mV) and (FeCoNiMoMn)_3_O_4_ (*η*_10_ = 362 mV, *η*_100 _= 416 mV). This is mainly attributed to the fact that Ru has a greater electronegativity than other metals, which acted as an electron acceptor to promote the nucleophilic reaction of H_2_O. In addition, the formed abundant O vacancies on the surface of (FeCoNiMoRu)_3_O_4_ also promoted the OER. 3D HEMs present a hierarchical architecture that integrates nanoscale porosity with interconnected networks, promoting enhanced reactant accessibility and electron transport. The high‐entropy Ir‐Ru‐based oxide RuIrFeCoNiO_2_ assembled from nanosheets exhibited excellent OER performance in acidic media (*η*_10_ = 189 mV), which is superior to those of commercial RuO_2_ (*η*_10_ = 253 mV) and C‐Ir/C (*η*_10_ = 292 mV).^[^ ^242^ ^]^ The 3D interconnected stacked nanosheet structure significantly increased the specific surface area and effectively exposed active sites.^[^ ^243^, ^244^ ^]^ Under multi‐element modulation, Ru as the active sites efficiently transfer electrons to surrounding metal elements, which increases the bonding state of the metal d state and the oxygen 2p state and reduces the antibonding states effectively promoting the stability of the RuIrFeCoNiO_2_ structure. Figure 12j shows the valence cloud of the metal atom d state and the oxygen atom p state. Additionally, the acid‐resistant precious metal Ir, along with transition metals Fe, Co, and Ni, further stabilizes lattice oxygen, shifting the oxygen evolution pathway toward the AEM mechanism.

#### 4.2.2 Pristine Non‐Noble Metal‐Based HEMs

**Figure 13:**
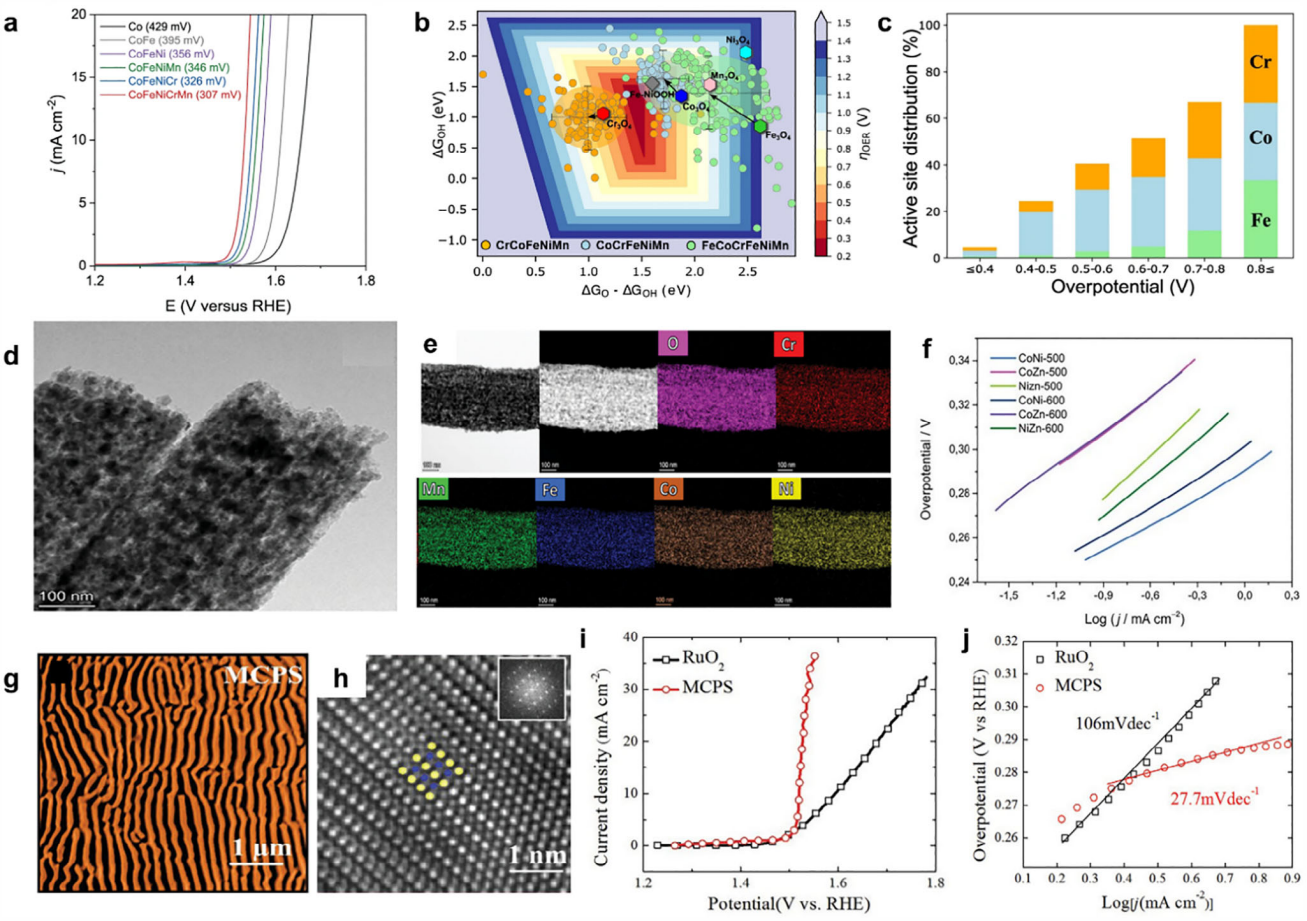
a) OER polarization curves normalized to geometric area in 1 m KOH electrolyte. b) Theoretical OER overpotentials are presented as 2D volcano plot heatmaps based on first‐principles calculations of *O and *OH binding energies for HEO, with the *OOH energy constrained. c) Dynamic redistribution of Cr, Co, and Fe active sites in HEO under electrochemical OER conditions. a–c) Reproduced under the terms of the CC‐BY Creative Commons Attribution 4.0 International license.^[^ ^25^ ^]^ Copyright 2023, Springer Nature. d) TEM and e) HAADF‐STEM images of (CrMnFeCoNi)_3_O_4_. f) Tafel curves of (CrMnFeCoNi)_3_O_4_ and comparison materials. d–f) Reproduced with permission.^[^ ^245^ ^]^ Copyright 2023, Wiley‐VCH. g,h) SEM and fast Fourier transformation (FFT)‐filtered HRTEM of MCPS. i) OER polarization curves, j) Tafel curves of MCPS and RuO_2_ in 0.1 m KOH. g–j) Reproduced with permission.^[^ ^55^ ^]^ Copyright 2020, Wiley‐VCH.

Transition metal‐based catalysts have shown great potential for OER. Non‐noble metal HEMs composed of multiple transition metals will synergistically regulate the d orbital electrons, redistribute the charge, and thus reduce the catalytic reaction energy barrier. The electronic properties of HEMs can be effectively tuned through strategic element selection, with the diverse range of transition metals offering abundant opportunities for designing efficient OER catalysts. At present, elements such as Fe, Co, Ni, and Mn show inherent performance for OER, but their catalytic activity remains unsatisfactory. Therefore, with metals such as Fe, Co, Ni, and Mn as the main elements, the introduction of other non‐precious metal elements (Cr, Cu, and Ti, etc.) and the design of nanoscale (0D, 1D, 2D, and 3D) structures will further enhance the OER performance. For instance, FeNiCoCrMnV HEO 0D nanoparticles with a diameter range of 20–80 nm were in situ synthesized on carbon fibers for efficient OER. In FeNiCoCrMnV HEO, Fe, Co, and Ni are active sites for OER, and Cr, Mn, and V optimize the oxidation state of the active metals to improve the intrinsic activity of the active sites.^[^ ^170^ ^]^ The introduction of Cr can also maximize the surface adsorbed highly oxidized species and surface oxygen species (O_2_^2−^/O^−^), which are often regarded as descriptors of enhanced OER activity. Thus, FeNiCoCrMnV HEO achieved high alkaline OER activity with an overpotential of 247 mV at 10 mA cm^−2^. Moreover, the spinel‐type HEO incorporating five metal cations (Co, Fe, Ni, Cr, and Mn) also demonstrated excellent alkaline OER performance with a low overpotential of only 307 mV at 10 mA cm^−2^ (**Figure** **13**a).^[^ ^25^ ^]^ DFT calculations of *OH binding energies at different active sites revealed that Co sites exhibited the lowest theoretical overpotential (0.29 V), contributing most significantly to the overall OER activity, while Cr and Fe sites showed moderately lower overpotentials (both 0.34 V) with secondary contributions (Figure 13b,c). Notably, the variation in adsorption energies at active sites correlates strongly with local strain effects. For instance, Fe neighbors expand Co─O bonds and strengthen *O/*OH binding, while Mn and Cr neighbors cause bond contraction and weaken their adsorption. These results highlight how tuning atomic radii of surrounding metals can modulate adsorption energies of the active sites, offering atomic‐level insight into the structure–activity relationship in HEO catalysts.

Similar to noble metal‐based HEMs, transitioning from nanoparticles to higher‐dimensional structures (1D, 2D, and 3D) further accelerates the reaction kinetics of HEMs for OER. 1D (CrMnFeCoNi)_3_O_4_ (CN‐NFs) spinel‐structured oxide nanofibers obtained by electrospinning are composed of polyhedral nanoparticles, resulting in abundant pores on their surfaces (Figure 13d,e).^[^ ^245^ ^]^ The (CrMnFeCoNi)_3_O_4_ (CN‐NFs) spinel oxide exhibited a lower Tafel slope of 59.6 mV dec^−1^ than those of 62.5 mV dec^−1^ of (CrMnFeCoZn)_3_O_4_ (CZ‐NFs) and 59.6 mV dec^−1^ of (CrMnFeNiZn)_3_O_4_ (NZ‐NFs) when replacing Ni or Co by Zn for alkaline OER, which is mainly due to the larger radius of Zn^2+^ ions leading to the destruction of the tetrahedral structure and reducing the OER activity (Figure 13f). In recent years, 2D nanosheet materials represented by transition metal (oxy) hydroxide have been selected as OER catalysts. Cross‐linked CoCuFeMoOOH nanosheets (thickness of ≈1.5 nm) exhibited a lower reaction barrier (1.735 eV), stronger metal‐O covalency and O─H bond‐breaking ability compared with ternary CoCuMoOOH and CoFeMoOOH.^[^ ^41^ ^]^ The overpotential of CoCuFeMoOOH is 199 mV (*η*_10_) for alkaline OER, which is better than that of CoFeMoOOH (*η*_10_ = 253 mV) and CoCuMoOOH (*η*_10_ = 273 mV). The superior activity is mainly attributed to the short‐range ordered structure and the increase of high‐valent Co^3+^ species in quaternary CoCuFeMoOOH HEMs. 3D high‐entropy structures based on non‐precious metals combine high porosity with interconnected conductive networks, maximizing mass and charge transport for efficient OER. A high‐entropy metal hydroxide‐organic framework (HE‐MHOF) demonstrated exceptional OER performance.^[^ ^246^ ^]^ The 3D architecture of HE‐MHOF provided abundant active sites for OER, where the metal cations coordinated with hydroxyl groups and organic linkers create a highly dispersed active site distribution. Remarkably, the HE‐MHOF achieved a current density of 100 mA cm^−2^ at a low potential of ≈1.64 V versus RHE. Structural analysis showed that the HE‐MHOF retains a Ni‐MOF‐like framework but features lattice distortion from uniformly distributed multi‐metal cations, forming a stable 3D structure and enabling over 100 h of stable operation at 10 mA cm^−2^.

#### 4.2.3 High Entropy Single Atoms Catalysts

HESACs possess a high density of active sites, breaking the linear scale between the catalytic activity and metal loading of conventional single‐atom catalysts (SACs). In addition, the distance between adjacent metal atoms in HESACs is close enough to be interconnected, which is conducive to multi‐electron coupling and effective diffusion of reactants, making it suitable for catalyzing OER.^[^ ^247^ ^]^ Under the evaluation of DFT and machine learning (ML), a variety of metal atoms were screened on the surface of nitrogen‐doped graphene, and the obtained FeCoNiRu‐HESACs showed the highest OER activity.^[^ ^248^ ^]^ Specifically, Co atoms acted as active sites for OER, while Fe, Ni, and Ru sites efficiently promoted the intrinsic catalytic activity of Co sites by changing the d orbital electron transfer through non‐bonded interactions. The results showed that the formation of *O intermediates is the RDS of the entire OER in FeCoNiRu‐HESAC, with a theoretical overpotential of 0.28 V.

#### 4.2.4 High Entropy Intermetallic

Multiple active sites and specific atomic arrangements in HEI facilitate OER through stepwise reactions at different sites. In recent years, HEIs with ordered structures such as L1_2_, B2, Heusler, Laves, and C40 have been synthesized and applied in different fields. Unlike the FCC solid solution, the ordered Laves phase has strong corrosion resistance. Taking advantage of this feature, Laves phase Fe‐Co‐Ni‐Cr‐Nb HEI (MCPS) with a layered porous structure has been prepared by selectively etching eutectic precursors (FCC and Laves) (Figure 13g,h).^[^ ^55^ ^]^ As shown in Figure 13i,j, MCPS shows a low overpotential (*η*_10_ = 0.288 V) and Tafel slope (27.7 mV dec^−1^), which is better than RuO_2_ (*η*_10_ = 0.384 V, Tafel slope = 106 mV dec^−1^). The presence of abundant oxides and hydroxides on the surface of MCPS could reduce the OER energy barrier. In addition, Fe, Co, Ni, and Cr were oxidized to higher valence states during the OER process, which is conducive to the absorption of polarized molecules to promote OER. Nb was reduced during the OER process (Nb^5+^→Nb^4+^), which is conducive to local charge transfer. This local oxidation/reduction and ordered multi‐active sites in MCPS promote the OER process.

#### 4.2.5 Defective HEMs

**Figure 14:**
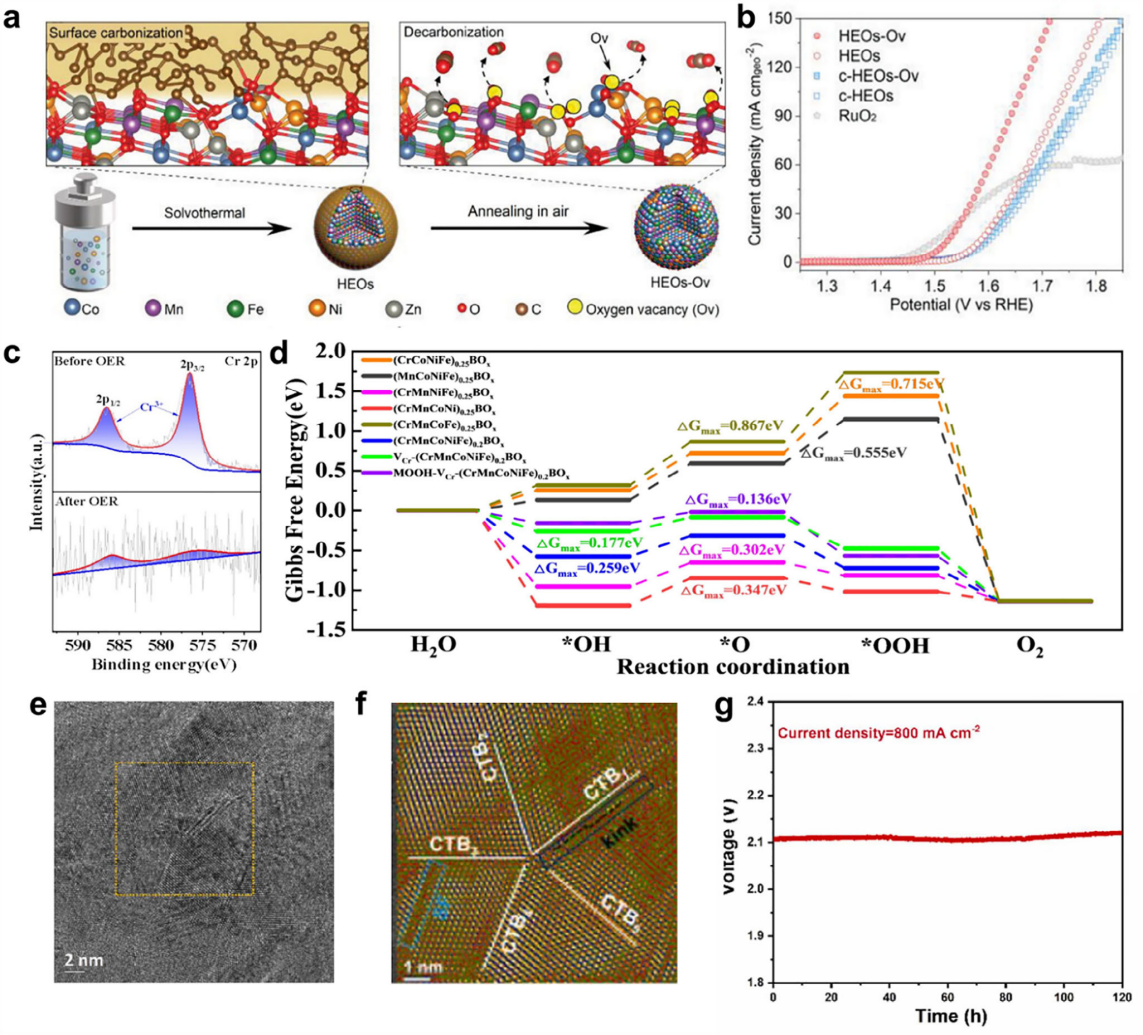
a) Schematic diagram for the synthesis of CoMnFeNiZn and CoMnFeNiZn‐O_v_. b) OER LSV curves of CoMnFeNiZn‐O_v_ and other comparative samples. a,b) Reproduced with permission.^[^ ^143^ ^]^ Copyright 2024, Wiley‐VCH. c) X‐ray Photoelectron Spectroscopy of Cr 2p in (CrMnCoNiFe)_0.2_BO_x_ before and after OER reaction. d) OER Gibbs free energy diagram of (CrMnCoNiFe)_0.2_BO_x_ and comparative catalysts. c,d) Reproduced with permission.^[^ ^180^ ^]^ Copyright 2024, Elsevier. e) HRTEM image of FeCoNiCuMoB HEA. f) (Inverse) FFT image corresponding to the selected area in (e). g) Stability test of FeCoNiCuMoB HEA in 1 m KOH at 800 mA cm^−2^. e–g) Reproduced with permission.^[^ ^251^ ^]^ Copyright 2025, Elsevier.

Defect engineering plays a crucial role in enhancing the OER activity of HEMs by modulating electronic structure, facilitating electron transport, and optimizing the adsorption energies of key reaction intermediates (*O, *OH, *OOH), thereby improving catalytic performance. Various types of defects can be strategically introduced into HEMs to boost OER performance.^[^ ^249^, ^250^ ^]^ Vacancy defects (e.g., oxygen and metal vacancies) create unsaturated coordination sites that enhance catalytic activity, while structural defects like dislocations and grain boundaries alter the local coordination environment to facilitate reaction kinetics. By precisely designing these defects, a synergistic effect among multiple elements can be achieved, thereby optimizing the activity and stability of HEMs for practical OER applications. Spinel‐type HEO nanofibers rich in oxygen vacancies (O_Vs_) have been prepared via electrospinning and low‐temperature calcination.^[^ ^245^ ^]^ Low‐temperature treatment induces a transformation from tetrahedral to octahedral coordination in cations, leading to the formation of O_Vs_ and coordinatively unsaturated metal octahedra (MO_6‐x_), which served as active sites for OER. Specifically, (Cr_1/5_Mn_1/5_Fe_1/5_Co_1/5_Ni_1/5_)_3_O_4_ HEO nanofibers calcined at 500 °C exhibited the best OER activity, with the lowest overpotential (360 mV for 10 mA cm^−2^) and Tafel slope (41 mV dec^−1^).^[^ ^143^ ^]^ Moreover, oxygen vacancies in HEOs can also be introduced through a low‐temperature surface carbonization‐decarbonization strategy (**Figure** **14**a). The resulting CoMnFeNiZn‐O_v_ not only promoted the pre‐oxidation process for faster OH^−^ adsorption but also enabled a unique bridge‐site pathway that facilitates easier deprotonation, thereby enhancing its OER performance (Figure 14b). Beyond oxygen vacancies, metal vacancies serve as equally critical structural features that modulate the electronic configuration and enhance the intrinsic catalytic performance of high‐entropy materials. The high entropy metal borate (CrMnCoNiFe)_0.2_BO_x_ generated Cr vacancies during the OER process (Figure 14c), which effectively promoted the charge transfer between OER intermediates and adjusts the adsorption strength.^[^ ^180^ ^]^ In addition, unlike other quaternary metal oxides, the RDS of (CrMnCoNiFe)_0.2_BO_x_ changed from the *O→*OOH step to the *OH→*O step, and the reaction energy barrier decreases from 0.302–0.867 eV to 0.259 eV, which shows that HEMs can adjust the RDS and thus enhance the OER activity (Figure 14d). In alkaline media, (CrMnCoNiFe)_0.2_BO_x_ exhibited excellent OER performance with an overpotential of only 236 mV (*η*_10_) at 10 mA cm^−2^ and a Tafel slope of 64 mV dec^−1^, which is better than (MnCoNiFe)_0.25_BO_x_ (*η*_10 _= 267 mV, 90.6 mV cm^−1^), (CrCoNiFe)_0.25_BO_x_ (*η*_10_ = 267 mV, 99.3 mV cm^−1^), (CrMnNiFe)_0.25_BO_x_ (*η*_10_ = 253 mV, 99.8 mV cm^−1^), (CrMnCoNi)_0.25_BO_x_ (*η*_10_ = 253 mV, 84.9 mV cm^−1^), and (CrMnCoFe)_0.25_BO_x_ (*η*_10_ = 256 mV, 102.8 mV cm^−1^).

Designing structural defect structures in HEMs can fine‐tune the atomic and electronic structures, thereby optimizing the adsorption behavior of OER intermediates. FeCoNiCuMoB HEA electrocatalysts with coherent twin boundaries were prepared on Ni foam (NF) by magnetron sputtering, in which abundant twin boundaries facilitated spontaneous electron migration and optimized the d‐band center, thereby strengthening the adsorption and activation of key OER intermediates.^[^ ^116^ ^]^ Moreover, interstitial B‐doped HEA films induce HEA films to exhibited a fivefold twin boundary structure, which optimized the surface electronic structure and improves the charge transfer kinetics (Figure 14e,f). As a result, the FeCoNiCuMoB HEA achieved an ultra‐low overpotential of 201 mV at 10 mA cm^−2^, and maintained long‐term stability (120 h) (Figure 14g). Similarly, CrMnFeCoNi HEA with twin defects (induced by Cr) and significant lattice distortions also showed efficient charge transfer and modulated electronic structure, achieving excellent OER performance with an overpotential of 265 mV at 10 mA cm^−2^ and a Tafel slope of 37.9 mV dec^−1^.^[^ ^251^ ^]^ DFT calculations revealed that these defects significantly lowered the energy barrier for the RDS of OER, particularly for the transition from *O to *OOH, thus boost the OER performance.

#### 4.2.6 Surface Reconstructed HEMs

Selective leaching or dissolution of surface elements can expose specific active sites, further boosting catalytic activity. During the OER process, the metals continuously leached from HEMs can lead to dynamic structural changes and further activation of active sites.^[^ ^252^, ^253^, ^254^, ^255^, ^256^, ^257^ ^]^ For example, relatively unstable elements (e.g., Mn, Cr, and V) in FeNiCoCrMnV HEO gradually leached out from the nanoparticles during the OER process.^[^ ^258^ ^]^ This leaching effect exposed more active sites (such as Fe, Ni, and Co) on the catalyst surface, increasing the number of available sites for binding reaction intermediates and thereby enhancing OER activity. Scanning transmission electron microscopy (STEM) observations confirmed significant morphological changes in FeNiCoCrMnV HEO, including the formation of smaller nanoparticles and SACs.^[^ ^141^ ^]^ The reconstructed FeNiCoCrMnV exhibited exceptional OER performance with an overpotential of only 220 mV at current density of 10 mA cm^−2^. Interestingly, the leaching process not only exposes additional active sites to improve OER activity but also facilitates self‐healing through surface migration, minimizing active site loss and enhancing stability. The introduction of high‐valent metals Mo and W effectively regulated the OER activity and stability of high‐entropy layered oxides FeCoMoW. The compensators Mo and W, and the high entropy properties of FeCoMoW themselves, repaired the loss of site Co^2+^. In borate buffer (KB_i_, pH = 14) with a small amount of Co^2+^ intentionally added, it can be maintained at 1.56 V versus RHE for up to 100 h.

Additionally, surface reconstruction has become a powerful method for tuning the structure and electronic properties of HEMs, significantly enhancing their catalytic performance. Under electrochemical conditions, HEMs, particularly HEAs and high‐entropy metal compounds, often undergo in situ surface oxidation or reduction, forming metastable (oxy)hydroxides or oxides that act as active sites for OER. Usually, the presence of Fe, Co, and Ni elements facilitates self‐reconstruction in OER to generate corresponding metal (oxy)hydroxides.^[^ ^259^, ^260^, ^261^, ^262^ ^]^ For example, (Ni_0.2_Co_0.2_Zn_0.2_Cu_0.2_Mg_0.2_) Fe_2_O_4_ nanofibers underwent surface reconstruction at a lower voltage and generated CoOOH, NiOOH, and FeOOH species with higher charge density, compared with other corresponding single, binary, ternary and quaternary oxides.^[^ ^170^ ^]^ These properties endow (Ni_0.2_Co_0.2_Zn_0.2_Cu_0.2_Mg_0.2_)Fe_2_O_4_ with superior alkaline OER activity with an overpotential of 286 mV for a current density of 10 mA cm^−2^, and the Tafel slope of 136 mV dec^−1^. Moreover, FeNiCoCrMnS_2_ not only gradually transformed from the initial yolk–shell structure to a porous sponge‐like morphology, but also continuously generated metal oxyhydroxides (MOOH) with increasing crystallinity during the OER process.^[^ ^90^ ^]^ In addition, the sulfides have been oxidized to generate sulfates (SO_4_^2^^−^) during electrochemical conditions, and the formed SO_4_^2−^ collaboratively with MOOH functioned as the real active centers for OER. The surface reconstructed FeNiCoCrMnS_2_ achieved current densities of 10, 100, 500, and 1000 mA cm^−2^ at overpotentials of 199, 246, 285, and 308 mV. It maintained 12 000 cycles and 55 h of continuous operation of 500 mA cm^−2^.

#### 4.2.7 Functionalized HEMs

**Figure 15:**
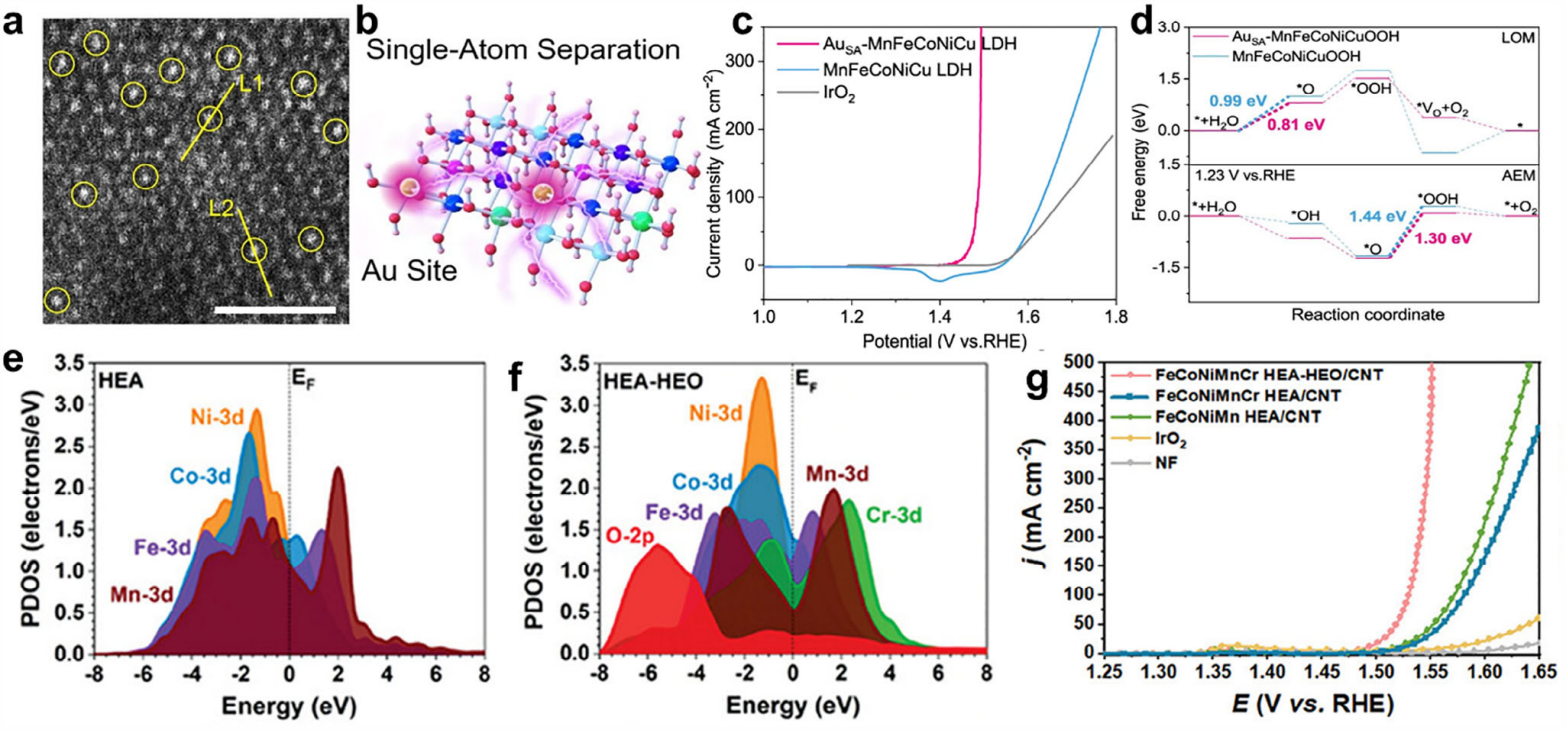
a,b) HAADF‐STEM image and atomic structure schematics of Au_SA_‐MnFeCoNiCu LDH. c) Polarization curves of Au_SA_‐MnFeCoNiCu LDH, MnFeCoNiCu LDH and IrO_2_. d) Free energy of Au_SA_‐MnFeCoNiCu LDH and MnFeCoNiCu LDH. a–d) Reproduced under the terms of the CC‐BY Creative Commons Attribution 4.0 International license.^[^ ^24^ ^]^ Copyright 2020, Springer Nature. e,f) PDOS of HEA and HEA‐HEO. g) Polarization curves of HEA‐HEO and other comparative catalysts. e–g) Reproduced with permission.^[^ ^149^ ^]^ Copyright 2024, Wiley‐VCH.

Similar to HER, further functionalizing HEMs with single‐atom doping, metal clusters, heterostructure formation, surface modifications, and hybridization with conductive supports can further optimize their OER catalytic properties.^[^ ^263^, ^264^, ^265^, ^266^, ^267^ ^]^ These strategies introduce cocktail effects that enhance active site exposure, tune electronic structures, and improve reaction kinetics, significantly boosting catalytic efficiency and stability. Single‐atom incorporation into HEMs offers a powerful strategy for optimizing OER performance by fundamentally altering the catalytic mechanism. For instance, atomically dispersed Au on high‐entropy MnFeCoNiCu LDHs, achieved a current density of 10 mA cm^−2^ at an overpotential of only 213 mV for alkaline OER, which is 110 mV lower than that of the pristine MnFeCoNiCu LDH (**Figure** **15**a–c).^[^ ^24^ ^]^ The enhanced activity is ascribed to the high electronegativity of Au single atoms, which strengthened the metal‐oxygen covalency, facilitated lattice oxygen activation, and triggered the LOM, thereby reducing the OER reaction energy barrier (Figure 15d). Furthermore, the high‐entropy effect enhanced the structural stability by suppressing metal dissolution with the Au_SA_‐MnFeCoNiCu LDH showing only a 6.4% drop in current density over 700 h, confirming excellent long‐term durability. Another approach is to introduce atomic clusters onto the HEM surface, which will optimize the interaction of HEM with OER active intermediates. A chemical dealloying strategy was used to directly modify the surface of (AlCoFeMoCr)_3_O_4_ spinel HEO with a diameter of ≈1.5 nm (np‐HEO/Pt).^[^ ^268^ ^]^ The surface‐distributed Pt clusters not only improved the electronic conductivity of HEO/Pt but also promoted the formation of *OOH active intermediates and the desorption of oxygen molecules. In addition, the doping of Mo and Cr made the number of e_g_ electrons of CoFe at the active site closest to 1, significantly increasing the OER activity. In 1 m KOH solution, the overpotential of np‐HEO/Pt at 10 mA cm^−2^ was ≈260 mV, which was better than np‐HEO (≈290 mV).

Constructing heterostructures in HEMs can stabilize active metal sites via heterointerfaces, thereby enhancing the OER activity and stability.^[^ ^222^, ^269^, ^270^, ^271^, ^272^, ^273^ ^]^ For example, FeCoNiMnCr nanoparticles with heterostructured HEA and HEO dispersed on carbon nanotubes (FeCoNiMnCr HEA‐HEO/CNT) were in situ synthesized via a Cr‐induced self‐reconstruction strategy.^[^ ^149^ ^]^ HEO formation in the heterostructure weakens the overlap of 3d orbitals, shifting the 3d orbitals of Ni and Co closer to the Fermi level, thereby increasing electron density and OER catalytic activity (Figure 15e,f). Simultaneously, the modulation of Fe, Mn, and Cr 3d orbitals optimized the overall electronic structure, providing more active sites for OER. Strong HEA‐HEO interactions also enhance stability by preventing metal aggregation and disordered oxide shell formation. In 1 m KOH, FeCoNiMnCr HEA‐HEO/CNT achieved an overpotential of only 261 mV at 10 mA cm^−2^ and maintained a low overpotential of 320 V at a high current density of 500 mA cm^−2^ (Figure 15g).

Hybridizing HEMs with non‐metallic materials, such as carbon‐based supports or MOFs, can not only improve conductivity and stability but also tune the electronic structure of active sites.^[^ ^222^, ^274^ ^]^ FeCoNiAlMo/CNT composites were formed by anchoring HEA nanoparticles onto carbon nanotubes (CNTs), which enhanced electron transport and ensured uniform nanoparticle dispersion.^[^ ^179^ ^]^ The FeCoNiAlMo/CNT composite exhibited excellent alkaline OER activity, achieving a low overpotential of 311 mV at 100 mA cm^−2^. It maintained the activity without obvious degradation in a 12 h chronoamperometric test. MOF materials can provide synergistic effects and are considered to be good carriers of metal oxide nanoparticles. Loading HEMs on MOFs may obtain new physicochemical properties and further enhance OER. For example, the defect‐rich MnFeCoNiCu HEO NPs and porous MnFeCoNiCu‐MOF/NF (HE‐MOF) were composited to form HEO‐NPs@C@HE‐MOF. Structurally, this hybridization creates numerous oxygen vacancies and lattice distortions, providing active sites with unsaturated electronic structures that enhance the adsorption ability for OH^−^. Moreover, the porous structure of the HE‐MOF promotes mass transfer, prevents HEO nanoparticle agglomeration, and ensures overall stability during the OER process. In 1 m KOH solution, HEO‐NPs@C@HE‐MOF delivered a current density of 50 mA cm^−2^ at the overpotential of only 266 mV and also high stability at a current density of 50 mA cm^−2^ for 24 h.^[^ ^275^ ^]^ Additionally, coupling HEMs with metal compounds, including phosphides, sulfides, and nitrides, facilitates charge transfer and optimizes adsorption energies. The OER activity can be further improved by combining 2D MoS_2_ with HEMs, which have a large specific surface area and abundant active sites. 2D flower‐like 2H‐MoS_2_ was prepared on carbon cloth (MoS_2_‐CC) by the hydrothermal method, and amorphous FeCoNiMnCr HEA was uniformly deposited on MoS_2_‐CC (FeCoNiMnCr@MoS_2_‐CC) by electrodeposition technology. It is worth noting that 2H‐MoS_2_ underwent a redox reaction during the electrodeposition process, which strengthened the interaction between 2H‐MoS_2_ and HEA, allowing FeCoNiMnCr@MoS_2_‐CC to reach a current density of 10 mA cm^−2^ at an overpotential of only 210 mV in alkaline electrolyte. This is higher than FeCoNiMnCr‐CC (239 mV) and commercial RuO_2_ (323 mV).

## 5 Summary and Perspective

HEMs have emerged as a promising class of materials for catalyzing water splitting (HER and OER). With their unique multi‐element compositions and high configurational entropy, HEMs demonstrate several advantages in water splitting, such as tunable electronic structures, more accessible active sites, and enhanced stability. These properties address some of the critical challenges in water splitting for hydrogen production, where materials should be durable, efficient, and cost‐effective. This review aims to detail the evolution of HEMs from their structural foundations to functional applications in water splitting. The transition from single‐metal to multi‐component systems improved their chemical stability, enhanced intrinsic properties, and leveraged synergistic cocktail effects. The shift from random to more ordered atomic configurations has further optimized their catalytic performance. Additionally, the transformation from bulk materials to nanostructured forms has significantly exposed more active sites and improved mass transport. The development from pristine to reconstructed, defective, or functionalized HEMs marks a crucial advancement, enabling further tuning of their catalytic properties. Except for the structure and function evolution, this review also provides an in‐depth understanding of the roles each element plays for water splitting, which will provide a deep understanding of HEMs at the atomic level and also provide guidance for further design highly efficient HEM‐based electrocatalysts.

Despite their potential as promising catalysts for water splitting, the development of HEMs in electrocatalysis remains in its early stages, with significant challenges to address in the future.

### 5.1 Building a HEMs Database for Element Selection

Theoretical calculations (such as DFT, MD, ML, and ANN) can be used to build a HEMs database and open new opportunities, as the combination of five or more elements offers virtually limitless possibilities. By leveraging emerging high‐throughput techniques, it becomes possible to rapidly generate and evaluate a vast number of HEM specimens, significantly accelerating materials discovery and development. For example, selecting elements for a specific reaction can start by evaluating their electronic structure within HEMs, such as the d‐band center, density of states (DOS), and charge transfer characteristics. It is also important to consider electron transfer between the selected elements, as this can lead to surface charge redistribution in the material and thus affect the adsorption of reactants/intermediates. Additionally, the compatibility of elements can be also assessed using theoretical calculations, helping to avoid a time‐consuming “trial‐and‐error” approach and determine whether a single solid solution phase can form under given conditions. Such a database not only streamlines material selection and optimization but also opens up exciting opportunities for uncovering new materials with enhanced performance for electrocatalysis.

### 5.2 The Scalability of HEM Synthesis Methods

To enable the practical use of HEMs for hydrogen production via water splitting, it is essential to develop synthesis strategies that offer moderate to high productivity. Currently, the fabrication of HEMs mostly involves harsh conditions, including high temperature, rapid quenching, or high pressure, significantly limiting their scalability for large‐scale catalyst production. Importantly, the development of advanced synthetic strategies can enable precise control over morphology, surface area, and porosity, thereby exposing more active sites and enhancing catalytic performance. Wet‐chemical routes such as sol–gel synthesis, co‐precipitation, and hydrothermal or solvothermal methods hold great promise for large‐scale production, offering fine control over composition, morphology, and porosity at relatively low temperatures. In addition, techniques like spray drying and mechanochemical synthesis could be considered for HEM synthesis at an industrial scale due to their operational simplicity and compatibility with continuous manufacturing processes. Moreover, green chemistry principles, such as using recyclable precursors, and energy‐efficient heating techniques like microwave or ultrasound‐assisted synthesis, may also be considered when preparing HEMs to enhance the sustainability of the production process.

### 5.3 Electrodynamic Instability and Phase Separation

Stability remains a major challenge for HEMs, especially in harsh electrolytic environments for long‐term operation. The inherent compositional complexity that gives rise to their unique properties also leads to intricate phase behavior, which can result in the segregation of elements or the formation of undesired secondary phases. These phenomena can severely impact the structural integrity, activity, and durability of HEM‐based catalysts. The integration of computational methods, high‐throughput experimentation, and in situ/operando characterization tools will be critical in unraveling the complex interplay between entropy, enthalpy, and reaction environment to understand the catalytic decay mechanism. Exploring the limits of phase stability and understanding how phase transitions influence the local structure and catalytic performance of HEMs will contribute to the rational design of more robust materials for electrocatalysis.

### 5.4 Identification of Active Centers

The unique multi‐element nature of HEMs creates a vast array of potential active sites with varying atomic arrangements and chemical environments. However, the individual contributions of each element to the overall catalytic performance remain largely unclear. Moreover, active sites can evolve dynamically under reaction conditions for HEMs, especially for OER, which makes the real active phase to be re‐examined for water splitting. Therefore, identifying the real active sites in high‐entropy water‐splitting catalysts remains a significant challenge. Other than computationally evaluating the electronic structure, reaction intermediates, and reaction pathways, the development and utilization of HEM databases offer promising avenues to uncover active centers through novel descriptors and to establish new mechanistic insights. Operando and in situ characterization techniques are strongly recommended to deepen our understanding of catalytic behavior in HEMs. Moreover, the highly diverse surface sites induced ensemble effect should also carefully be considered when determining the active sites, as each site may exhibit different sensitivities to elementary reaction steps.

### 5.5 Integration with Advanced Electrolyzer Systems

While the structural advantages of HEMs have accelerated their development in water splitting, their practical application is still in its early stages. The integration of HEMs into advanced electrolyzer systems holds great promise for enhancing catalytic efficiency and long‐term durability. However, it also presents challenges in the scalability of synthesis methods, electrochemical stability, compatibility with existing electrolyzer components, and performance predictability due to the multicomponent nature of HEMs. Developing standardized synthesis protocols, robust computational tools for property prediction, and advanced in situ characterization techniques to monitor material behavior under operating conditions will advance the integration of HEMs in water electrolyzer systems. Moreover, cross‐disciplinary approaches that integrate materials science, electrochemical engineering, and data‐driven design will be also essential to accelerate next‐generation electrolyzer technologies with HEMs for sustainable hydrogen production.

**Table 1:** Summary of HEMs for the HER.

Strategies	Catalysts	Electrolyte	Overpotential [mV] for 10 mA cm^−2^	Tafel slope [mV dec^−1^]	Refs.
0D HEMs	PdPtRuRhAu HEAs	0.5 m H_2_SO_4_	70.07	30.3	[163]
	Pt‐Pd‐Ni‐Co‐Mn	1 m KOH	22.6	77.5	[201]
	Pt_18_Ni_26_Fe_15_Co_14_Cu_27_/C	1 m KOH	11	30	[44]
	PtCoNiRuIr/C	0.5 m H_2_SO_4_	18	34.2	[87]
	PtPdIrRhAuAgCu‐rEGO	1 m KOH	11.3	59.9	[95]
	NiCoMoPtRu HEANCs	1 m KOH	9.5	29.8	[52]
	Pt4FeCoCuNi	1 m KOH	20	31	[51]
	Ru–PtFeNiCuW/CNTs	1 m KOH	16	27	[50]
	PtRuPdCoNi HEA	0.5 m H_2_SO_4_	16	27	[84]
	PtRuMoFeCoNi	0.5 m H_2_SO_4_	11	28.7	[86]
	PdFeCoNiCu/C	1 m KOH	18	39	[146]
	PtPdCoNiMn	1 m KOH	48.7	20.7	[96]
	PtPdCoNiMn	1 m KOH	13	29.6	[91]
	PtFeCoNiIr/C	0.5 m H_2_SO_4_	20.3	12.9	[99]
	NiCoFeMoMn@6h	1 m KOH	14	29	[208]
	FeCoNiCuMn HEA	1 m KOH	281 (100)	53	[110]
	FeCoNiCuMo	6 m KOH	68	100	[97]
	NiCoFeMnCrP	1 m KOH	253	94.5	[98]
1D HEMs	Al−Ni−Co−Ru−Mo	1 m KOH	24.5	30.3	[204]
	PtNiCoFeMo	0.5 m H_2_SO_4_	42(100)	21.2	[203]
	CuNiCoRuIr HEA NT	1 m KOH	22	69	[202]
	CoZnCdCuMnS@CF	1 m KOH	173	93.4	[114]
	FeCoNiCuMnN/CC‐400	1 m KOH	184	113	[111]
2D HEMs	PdMoGaInNi	0.5 m H_2_SO_4_	13	127.6	[205]
	Pt/HE‐LDH	0.5 m H_2_SO_4_	42	42	[112]
	Co_0.6_(VMnNiZn)_0.4_PS_3_	1 m KOH	65.9	65.5	[88]
	CrFeCoNiZn HES	0.5 m H_2_SO_4_	15	105	[92]
	(MoWNbTaV)S_2_	0.5 m H_2_SO_4_	84	90	[93]
	FeNiCoMnVOx	1 m KOH	81	88	[209]
	(FeMnMoNi)Se_2_	1 m KOH	30.5	49.6	[210]
	FeCoNiCuP	1 m KOH	113	52.4	[34]
3D HEMs	PtPdRuMoNi‐HEA	0.1 m KOH	25	38	[175]
	PtFeCoNi@HCS	1 m KOH	29	136.42	[190]
	Pt‐Pd‐Ni‐Co‐Mn HEA	1 m KOH	43.7	75.5	[184]
	FeCoNiRu‐450	1 m KOH	40	84	[201]
	PtMoPdRhNi	1 m KOH	9.7	25.9	[107]
	PtFeCoNiCu HEA	0.5 m H_2_SO_4_	30.7	28.1	[166]
	Pt_34_Fe_5_Ni_20_Cu_31_Mo_9_Ru	0.5 m H_2_SO_4_	9	27	[185]
	Pt_26_Ir_7_Fe_13_Co_22_Ni_32_	1 m KOH	29	44.5	[113]
	NiCoMoZnCu HEANFA	1 m KOH	242.9(500)	61.4	[161]
	V_1.0_CuCoNiFeMn	1 m KOH	50(50)	148	[212]
	NNM‐HEA@CF	1 m KOH	85.2(100)	86.3	[168]
	H‐FeCoNiCuMo	1 m KOH	21	18.5	[173]
	Mil53 MOF	1 m KOH	206	118.7	[211]
	CoFeNiCrMnP/NF	1 m KOH	51(100)	48	[36]
	HEOC	0.5 m H_2_SO_4_	57	34.6	[33]
Defect	(MoWVNbTa)C	0.5 m H_2_SO_4_	156	78	[100]
	(FeCoNiB_0.75_)_97_Pt_3_	1 m KOH	27	30.9	[216]
	IrRuRhMoW HEA	0.1 m KOH	28	51	[217]
	Pt(Co/Ni)MoPdRh HEAs	1 m KOH	16.5	26.8	[58]
	HEA‐PdPtRhIrCu	1 m KOH	15	37	[142]
	CoNiCuZnFeP	1 m KOH	318	121.3	[189]
	PdFeCoNiCu‐pHENs	1 m KOH	38.4	29	[215]
HESAs	PtIrCuNiCo	1 m KOH	22	/	[213]
	PtRuRhPdRe‐MoSe_2_	1 m KOH	35	90	[106]
Ordered HEMs	Pt_4_FeCoCuNi PCPAF‐HEA/C	1 m KOH 0.5 m H_2_SO_4_	20 24	31 29	[51, 214]
	FeCoNiAlTi	1 m KOH	88.2	40.1	[43]
	NiCoFeMoMn	1 m KOH	14	29	[208]
	FeCoNiMnRu/CNFs	1 m KOH	/	67.4	[121]
	HEI_800/C	1 m KOH	128	21	[122]
	(FeCoNi)(RuPt) HEI	1 m KOH	/	47.1	[127]
	(CoNiRhIrRu)Sb_3_	0.5 m H_2_SO_4_	200	/	[128]
	PtRuFeCoNi	0.5 m H_2_SO_4_	41.3	/	[123]
	FeCoNiMnMoP	1 m KOH	55	65.2	[129]
Reconstructive HEMs	PtRu_2.9_Fe_0.15_Co_1.5_Ni_1.3_ FeCoNiRu‐450 NiFeCrVTi HEA	1 m KOH 1 m KOH 3.5% NaCl	11.8 40 37.9	26.3 84 36.2	[107, 181, 218]
	AlCoCrFeNi	1 m KOH		92.89	[133]
	CNFMPO	1 m KOH	43	33.5	[134]
	NiCoMoZnCu/CFC	1 m KOH	/	61.4	[212]
	FeCoNiVCrZn HEA	1 m KOH	249	50	[135]
	FeCoNiCuPd HEA	1 m KOH	29.7	47.2	[109]
Functionalized HEMs	HEA@Pt Pt/HE‐LDH	1 m KOH 0.5 m H_2_SO_4_	13.7 42	30.6 42	[112, 225]
	HEPi/C	0.5 m H_2_SO_4_	40	36	[119]
	Pt/(FeCoNiCrAl)_3_O_4_	1 m KOH	22	25.9	[228]
	Pt‐(LaCeSmYErGdYb)O	0.5 m H_2_SO_4_	12	10	[219]
	PtNiCuMnMo HEA	1 m KOH	44	74	[231]
	PtCoNiMoRh@Rh	0.5 m H_2_SO_4_	9.1	8.74	[232]
	MoS_2_@HEP	1 m KOH	71	58	[233]
	HEA/MoS_2_/MoP	1 m KOH	148	71.98	[270]
	PtRhCoNiCu/CC	1 m KOH	19	26.9	[222]
	V‐Co_2_P@HE	1 m KOH	33	47.44	[271]
	NiMoCoMnLa@Ni	1 m KOH	146	79	[226]
	HEA/CNT‐10	1 m KOH	30.7	71	[191]
	MoS_2_@HEP	1 m KOH	71	58	[233]
	FeCoNiWCuOOH@Cu	1 m KOH	200	24	[230]
	AC‐HEA‐CuAgAuPtPd	0.5 m H_2_SO_4_	9.5	31	[220]
	NiFeCoZn/NiZn‐Ni/NF	1 m NaOH	/	46.58	[229]
	FeCoNiMnZn/N‐CNT	1 m KOH	184	112	[221]
	CoNiCuMgZn‐40@C	1 m KOH	158	36.1	[227]
	Fe_20_Co_20_Ni_20_Mo_20_Al_20_	1 m KOH	223	39.8	[192]
	(WNiCoMoRu)PO_x_/C	0.5 m H_2_SO_4_	40	36	[119]

**Table 2:** Summary of HEMs for the OER.

Strategies	Catalysts	Electrolyte	Overpotential [mV] for 10 mA cm^−2^	Tafel slope [mV dec^−1^]	Refs.
0D HEMs	RuMnFeMoCo	0.5 m H_2_SO_4_	170	49.7	[20]
	IrFeCoNiCu‐HEA	0.1 m HClO_4_	302	58	[164]
	(RuIrFeCoNi)O_2_	0.5 m H_2_SO_4_	261	56.35	[239]
	FeNiCuWRu	1 m KOH	267	32.4	[234]
	PtFeCoNiMnGa HEA	1 m KOH	243	40.2	[183]
	FeCoNiMnRuLa/CNT	1 m KOH	281	47.5	[235]
	IrRuNiMoCo	0.5 m H_2_SO_4_	243	56.2	[53]
	IrPdCuFeNiCoMo	0.5 m H_2_SO_4_	235	51	[27]
	FeCoNiPdW	1 m KOH	227	33	[182]
	(FeNiCoCrMnV) HEO	1 m KOH	247	45	[170]
	CoFeNiCrMn HEO	1 m KOH	307	34.7	[25]
	La(CrMnFeCo_2_Ni)O_3_	1 m KOH	325	51.2	[151]
	(CrMnFeCoNi)Sx	1 m KOH	295(100)	66	[29]
	FeCoNiMoW HEA	1 m KOH	233	36.7	[54]
	HEOH(FeNiCoCrMn)_Cl_	1 m KOH	250	/	[236]
	FeCoNiCuMn	1 m KOH	383(0.5)	/	[110]
	CoFeNiMnMoPi	1 m KOH	277.5	74	[237]
	FeCoNiCuMo‐O	1 m KOH	272	41	[170]
1D HEMs	AlNiCoRuMo	1 m KOH	250	54.5	[204]
	AlNiCoIrMo np‐HEA	0.5 m H_2_SO_4_	233	55.2	[160]
	FeCoNiCuPd	1 m KOH	194	39.8	[109]
	CrMnFeCoNi HESOs	1 m KOH	360	41	[245]
2D HEMs	(FeCoNiMoRu)_3_O_4_	1 m KOH	199	40	[241]
	CoCuFeAgRu	1 m KOH	280	70.7	[26]
	FeCoCuMnRuB	1 m KOH	233	61	[108]
	CoCuFeMoOOH@Cu	1 m KOH	199	48.8	[41]
	CoFeMnCuZn	1 m KOH	267	45	[89]
	CoFeNiMoWTe	0.5 m H_2_SO_4_	373	40.6	[187]
	FeCoMoW	1 m KOH	332	63.6	[90]
	(CrFeCoNiMo)_3_O_4_	1 m KOH	255.3	37	[32]
	FeCoNiWCuOOH@Cu	1 m KOH	200	23	[230]
	FeCuCoNiZn	1 m KOH	236	43	[240]
3D HEMs	M‐RuIrFeCoNiO_2_	0.5 m H_2_SO_4_	189	49	[85]
	(RuSnSbReF)Ox	0.5 m H_2_SO_4_	156	23.87	[177]
	PtPdFeCoNi/HOPNC	1 m KOH	310	88.7	[65]
	ZnNiCoIrMn	0.5 m H_2_SO_4_	237	46	[69]
	FeCoNiRu	1 m KOH	306	45	[107]
	MnCoNiCuZn	1 m NaOH	300	57	[246]
	NiFeCoMnOOH	1 m KOH	194	67.96	[171]
	FeCoNiMnMo	1 m KOH	279	56.1	[195]
	FeNiCo CrMnS2	1 m KOH	199	39.1	[141]
	FeCoNiMoCrOOH	1 m KOH	172	35.53	[64]
	MnFeCoNiVPS	1 m KOH	245	63.43	[197]
	ZnCoNiFeV	1 m KOH	253	49	[68]
	FeCoNiCuYP/C	1 m KOH	259	64	[67]
	HE‐MHOFs	1 m NaOH	410(100)	57	[246]
	FeNiCoCrMn	1 m KOH	229	40	[71]
	Ni‐HEA	1 m KOH	217	46.3	[243]
	FeCoNiMoMn	1 m KOH	218	53	[244]
Defect	CoFeMnCuZn	1 m KOH	267	45	[245]
	(CrMnCoNiFe)_0.2_BO_x_	1 m KOH	253	64	[180]
	FeCoNiCuMoB	1 m KOH	201	41.3	[116]
	CrMnFeCoNi	0.1 m KOH	265	37.9	[251]
	HEOs‐Ov	1 m KOH	284	53	[143]
	FeCoNiMnW@CCC	1 m KOH	253	41	[188]
	FeCoNiCrMn	1 m KOH	282	64.3	[249]
HESAs	FeCoNiRu‐HESAC	1 m KOH	280	/	[248]
	(FeCoNiCrCuAl)S@La	1 m KOH	253	51.75	[247]
Ordered HEMs	MCPS Al_0.5_NiCoCrMo_0.5_	0.1 m KOH 1 m KOH	288 327	27.7 168.91	[55, 130]
Reconstructive HEMs	FeNiCoCrXS_2_ (CoNiFeCuCr)Se_x_ FeCoNiRu‐450	1 m KOH 1 m KOH 1 m KOH	199 / 243	39.1 46.78 45	[107, 136, 141]
	FeNiCoCrMnV	1 m KOH	220	/	[170]
	NiFeCoMnAl	1 m KOH	190	47.62	[120]
	AlCoCrFeNi	1 m KOH	/	39.7	[133]
	FeCoNiCuPd/CFC	1 m KOH	194	39.8	[109]
	NiCoZnCuMg) Fe_2_O_4_	1 m KOH	286	136	[258]
	CNFMPO	1 m KOH	252	44.3	[134]
	*a*‐NiFeCoVMo	1 m KOH	172	/	[137]
	(FeCoMnZnMg)_3_O_4_	1 m KOH	240	59	[138]
	FeCoNiCrMn	1 m KOH	263	50.9	[139]
	FeCoNiCrVB	1 m KOH	237	24.2	[259]
	(VFeNiCoCu)_3_O_4_ HESO	1 m KOH	181	48.49	[260]
	HES/NF	1 m KOH	/	47.6	[261]
	FeCoNiZnOOH/NF	1 m KOH	/	49.2	[252]
	FeCoNiMnBPO_x_	1 m KOH	248	42.3	[253]
	FeCoNiCuMoOOH/NF	1 m KOH	201	39.4	[254]
	Ru_5_CoNiCuMn‐BDC	1 m KOH	240	33	[257]
	FeCoNiMnBO_x_	1 m KOH	266	64.5	[262]
	HEO‐P‐1	1 m KOH	254	47.4	[35]
	FCNMWO	1 m KOH	313	40.95	[255]
	(FeCoNiCuRu)S_2_	1 m KOH	193	46	[256]
	N_y_@ZrFeCoNiAlOSO_4_	1 m KOH	257	37.8	[273]
	FeCoNi_2_F_4_(OH)_4_	1 m KOH	298	56	[10]
Functionalized HEMs	CrZr‐HEA‐Rgo Au_SA_‐MnFeCoNiCu HE(Ru,Mo)‐MOF	0 m KOH 1 m KOH 1 m KOH	/ 213 267	75.33 27.5 36.3	[24, 182, 196]
	(FeCoNiCrCuAl)S@La	1 m KOH	246	51.75	[247]
	np‐HEO/Pt	1 m KOH	260	50.5	[268]
	Ag@CoCuFeAgMoOOH	1 m KOH	218	35.3	[147]
	FeCoNiMnCr	1 m KOH	261	42.2	[149]
	HEO‐NPs@C@HE‐MOF	1 m KOH	/	43.9	[274]
	(FeNiCuCoZn) _90‐x_V_20_P_10_	1 m KOH	228	23.6	[269]
	FeCoNiMnCr@MoS_2_‐C	1 m KOH	210	40.3	[275]
	HEA/MoS_2_/MoP	1 m KOH	230	63.54	[270]
	(CrFeCoNi)_97_O_3_	1 m KOH	196	29	[66]
	IrRuCoNiCu/CC	1 m KOH	166	69.1	[222]
	V‐Co_2_P@HE	1 m KOH	227	53.77	[271]
	MnCr_2_O_4_@O‐HEA	1 m KOH	268	51.6	[272]
	NiCo(FeCrCoNiAl _0.1_)Ox	1 m KOH	381	60.9	[263]
	FeCoNiMnCuP_x_/C	1 m KOH	239	72.5	[264]
	FeCoNiMo HEA‐MoC	1 m KOH	232	61.47	[265]
	FeCoNiMnCr HEA‐HEO	1 m KOH	255	37.3	[266]
	R‐SNCFCA_4.5_	1 m KOH	228	80.52	[267]
	HEO/Ti_3_C_2_T_x‐0.5_	1 m KOH	360	99	[193]

## Conflict of Interest

The authors declare no conflict of interest.
